# Bidirectional Mendelian Randomization of Causal Relationship between Inflammatory Cytokines and Different Pathological Types of Lung Cancer

**DOI:** 10.7150/jca.98301

**Published:** 2024-07-16

**Authors:** Xinhang Hu, Shouzhi Xie, Xuyang Yi, Yifan Ouyang, Wangcheng Zhao, Zhi Yang, Zhe Zhang, Li Wang, Xingchun Huang, Muyun Peng, Fenglei Yu

**Affiliations:** Department of Thoracic Surgery, Second Xiangya Hospital, Central South University, Changsha 410000, China.

**Keywords:** lung cancer, inflammatory cytokines, bidirectional Mendelian randomization

## Abstract

Prior research has proposed a potential association between lung cancer and inflammatory cytokines, yet the specific causal relationship remains unclear, especially across various lung cancer pathologies. This study utilized bidirectional Mendelian randomization (MR) to explore these causal connections, unveiling novel insights. Our research revealed distinctive inflammatory cytokine profiles for each subtype of lung cancer and identified potential biomarkers that could refine diagnostic and therapeutic approaches. We applied two-sample Mendelian randomization, leveraging genetic variance data from three extensive genome-wide association studies (GWAS) focusing on different lung cancer types (lung adenocarcinoma: 1590 cases and 314,193 controls of healthy individuals of European descent; lung squamous cell carcinoma: 1510 cases and 314,193 controls of European ancestry; small cell lung cancer: 717 cases and 314,193 controls of European ancestry). A separate GWAS summary on inflammatory cytokines from 8,293 healthy participants was also included. The inverse variance weighting method was utilized to examine causal relationships, with robustness confirmed through multiple sensitivity analyses, including MR-Egger, weighted median, and MR-PRESSO. Our analysis revealed that elevated levels of IL_1RA were associated with an increased risk of lung adenocarcinoma (OR: 1.29, 95% CI: 1.02-1.64, *p* = 0.031), while higher MCP_1_MCAF levels correlated with a decreased risk of lung squamous cell carcinoma (OR: 0.77, 95% CI: 0.61-0.98, *p* = 0.031). Furthermore, IL_10, IL_13, and TRAIL levels were positively associated with lung squamous cell carcinoma risk (IL_10: OR: 1.27, 95% CI: 1.06-1.53, *p* = 0.012; IL_13: OR: 1.15, 95% CI: 1.06-1.53, *p* = 0.036; TRAIL: OR: 1.15, 95% CI: 1.06-1.53, *p* = 0.043). No association was found between inflammatory cytokine levels and small cell lung cancer development, whereas SDF_1A and B-NGF were linked to an increased risk of this cancer type (SDF_1A: OR: 1.13, 95% CI: 1.05-1.21, *p* = 0.001; B-NGF: OR: 1.13, 95% CI: 1.01-1.27, *p* = 0.029). No significant relationship was observed between the 41 circulating inflammatory cytokines and lung adenocarcinoma or squamous cell carcinoma development. Our findings indicate distinct associations between specific inflammatory cytokines and different types of lung cancer. Elevated IL_1RA levels are a risk marker for lung adenocarcinoma, whereas higher MCP_1_MCAF levels appear protective against lung squamous cell carcinoma. Conversely, elevated levels of IL_10, IL_13, and TRAIL are linked with an increased risk of lung squamous cell carcinoma. The relationships of SDF_1A and B-NGF with small-cell lung cancer highlight the complexity of inflammatory markers in cancer development. This study provides a nuanced understanding of the role of inflammatory cytokines in lung cancer, underscoring their potential in refining diagnosis and treatment strategies.

## Introduction

Lung cancer remains the most prevalent cancer worldwide and is the primary cause of cancer-related deaths 1. It is mainly classified into two types: non-small cell lung cancer (NSCLC), which constitutes approximately 85% of all cases, and small-cell lung cancer (SCLC) 2. NSCLC is further categorized into subtypes such as lung squamous cell carcinoma, lung adenocarcinoma, and the less common large cell lung cancer 3. Despite advances in medical research, the overall five-year survival rate for lung cancer patients is still distressingly low, under 21% 4, 5. This low survival rate can be attributed largely to the lack of effective screening methods and the delayed diagnosis due to nonspecific symptoms, highlighting the critical need for improved diagnostic and therapeutic approaches 2.

Although smoking is a well-known risk factor, the underlying causes of lung cancer remain largely unknown. Recent advancements have shed light on the potential link between inflammation and lung cancer, with studies suggesting that inflammation within tumor tissues may play a significant role in cancer development, encompassing stages from initiation to metastasis 6. Chronic inflammatory conditions are associated with a heightened risk of several cancers, including those of the lung, liver, and colon 7. Inflammatory cytokines, crucial regulators of inflammation, are implicated in the progression of lung cancer. These cytokines serve dual functions: they activate immune responses to potentially curb tumor growth, but they can also facilitate the malignant transformation, proliferation, invasion, and spread of cancer cells 8. P In particular, IL-6 and IL-8 have been identified as risk factors for lung cancer among smokers 9, with TNF-α, IL-10, and IL-17 also being considered in various contexts 6, 10. The role of some cytokines, such as IL-17, in lung cancer remains contentious 11. Additionally, the precise causal relationships between changes in levels of inflammatory cytokines and the development or progression of lung cancer, as well as their interaction with pharmaceutical interventions, continue to be subjects of ongoing research and debate. To date, several studies have examined the causal link between inflammatory cytokines and lung cancer. For instance, Zhao Yang et al. proposed a causal relationship between certain interleukin (IL)-1 family members and lung cancer 12, while Emmanouil Bouras et al. associated IL-18 with lung cancer 7. However, our investigation reveals a gap in the literature concerning the exploration of causal relationships between different types of inflammatory cytokines and various pathological subtypes of lung cancer, as well as the absence of studies focusing on the potential variance in inflammatory cytokine profiles across distinct lung cancer pathologies. In our study, we employ a two-sample bidirectional Mendelian randomization (MR) approach to examine these causal associations and introduce new insights. Our objective is to delineate unique inflammatory cytokine profiles for each subtype of lung cancer and identify potential biomarkers to enhance noninvasive diagnostic and therapeutic strategies.

MR is a statistical approach that utilizes genetic variations from non-experimental data to infer causal relationships between exposures and outcomes, employing single nucleotide polymorphisms (SNPs) as genetic instrumental variables to represent those exposures 12-14. Unlike traditional observational studies, MR leverages alleles randomly assigned during meiosis to overcome confounding factors and reverse causality issues, providing a more reliable basis for causal inference 15, 16. In this study, we conducted a two-sample bidirectional MR analysis. We began by identifying effective genetic instrumental variables for 41 inflammatory markers using data from genome-wide association studies (GWAS). We then examined the relationship between these inflammatory markers and three different pathological types of lung cancer, further investigating the direction of causality through reverse MR analysis. This methodological approach helps lay the groundwork for the prevention of lung cancer via specific inflammatory cytokines, identifies potential therapeutic targets, and assists in differentiating between lung cancer types based on their inflammatory cytokine profiles.

## Materials and Methods

### Ethics and informed consent

The data for this study were obtained from four previously published GWAS. These studies were conducted in full compliance with ethical standards, having received the necessary approvals from relevant ethics committees. Additionally, all participants in these GWAS provided their informed consent in writing after being adequately informed about the research's objectives and procedures, ensuring the protection of the participants' rights, privacy, and well-being throughout the research process.

### MR hypothesis

**Figure 1:**
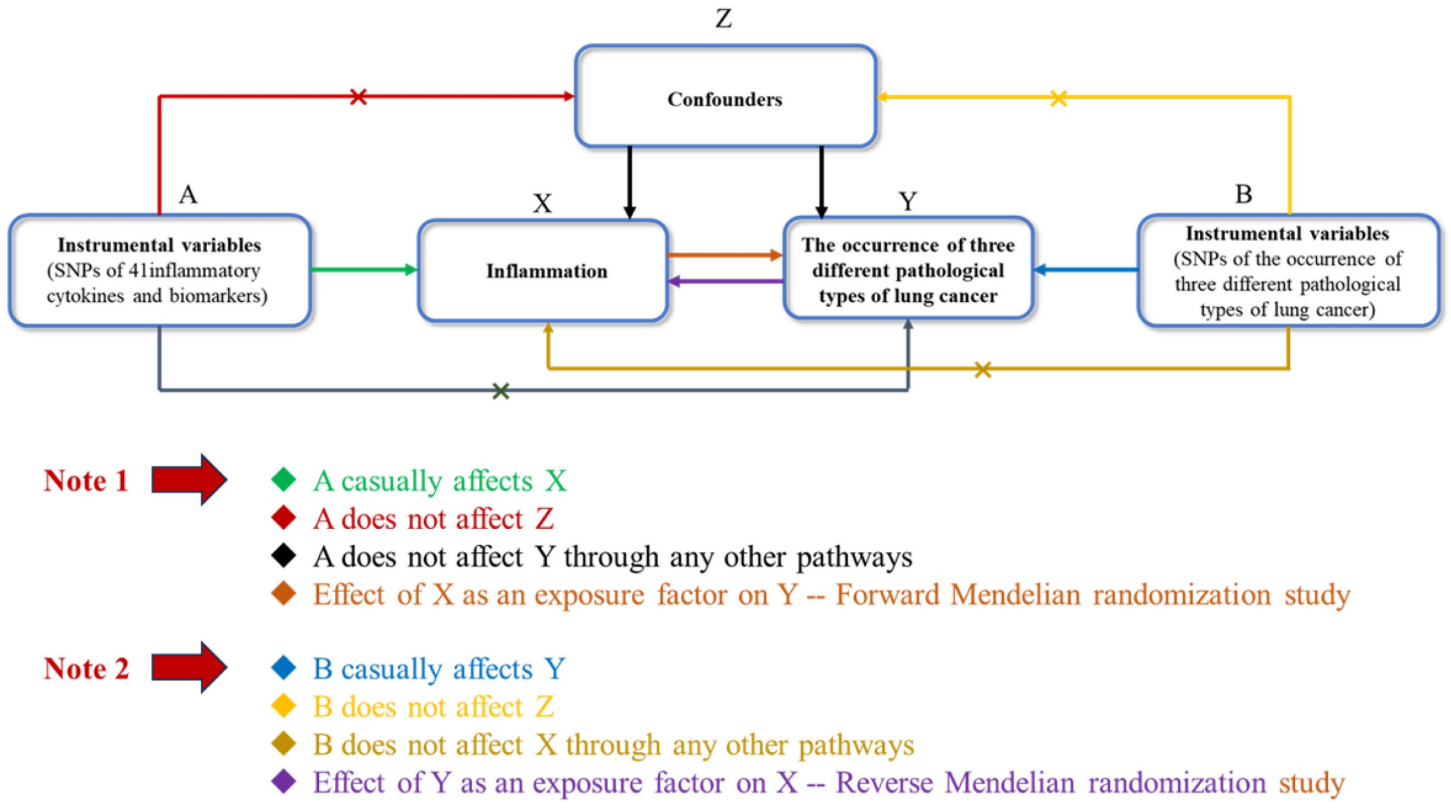
Bidirectional Two-Sample Mendelian Randomization Study Structure. This diagram illustrates the foundational principles necessary for the efficacy of Mendelian Randomization (MR) analysis, highlighting the critical assumptions: 1) Correlation, where genetic variants are significantly associated with the exposure; 2) Independence, ensuring genetic instruments are not linked to potential confounders; 3) Exclusion Restrictions, asserting that genetic variants influence the outcome exclusively through the exposure, without alternative pathways.

Figure 1 illustrates the structure and principles of our bidirectional two-sample MR study. The efficacy of MR analysis hinges on three fundamental assumptions:

1, Correlation: This principle requires that the genetic variants used as instruments must be significantly associated with the exposure of interest.

2, Independence: This criterion mandates that the genetic instruments must not be linked to potential confounders, ensuring their independence.

3, Exclusion restrictions: This assumption asserts that the genetic variants impact the outcome solely through their effect on the exposure, with no alternative pathways involved 18.

In our bidirectional study, we utilized data from four previously published GWAS to identify 41 significant SNPs associated with inflammatory cytokines and three types of lung cancer: SCLC, lung adenocarcinoma, and lung squamous cell carcinoma. Our methodological approach consisted of two primary phases. Initially, we identified genetic instrumental variables for each inflammatory cytokine to explore their potential causal relationships with SCLC, lung adenocarcinoma, and lung squamous cell carcinoma, respectively. Subsequently, we applied genetic instrumental variables related to SCLC, lung adenocarcinoma, and lung squamous cell carcinoma to examine the causal impact of these specific cancers on the levels of each circulating inflammatory cytokine. This stringent approach aligns with the principles of MR, offering a solid structure for evaluating the causal associations between inflammatory cytokines and various types of lung cancer.

### Selection of genetic instrumental variables

The selection of genetic instrumental variables in this study commenced with a strict threshold set at p < 5 × 10-8, identifying SNPs strongly linked with lung cancer and inflammatory cytokines. When few SNPs were found for certain cytokines 19, a slightly relaxed threshold of p < 5 × 10-6 was applied 19. Following this, we ensured that instrumental variables were independent by removing any with linkage disequilibrium (r² < 0.001 within 10,000 kb) 20. Each SNP's contribution to explaining variance in the exposure was determined using the R² value, and the F-statistic was used to identify weak instrumental variable bias, with an F > 10 suggesting no significant bias 21. Additionally, we utilized the PhenoScanner database, applying a genome-wide significance threshold (p < 5 × 10-8) to identify SNPs related to potential confounders such as smoking habits or specific outcomes. SNPs associated with such confounders were subsequently excluded from the MR analysis to ensure the integrity and reliability of the results.

### Data sources

In this study, we utilized data from four publicly available GWAS summary datasets. The three GWAS summaries for lung cancer were sourced from the 10th generation Finnish database, categorizing lung cancer as LUAD, LUSC, and other types based on the international classification of diseases for oncology (ICD-10 or ICD-O-3). It is important to note that all lung cancer diagnoses underwent confirmation through histopathological analysis. The dataset for SCLC was extracted from the C3_SCLC_EXALLC section of the R10_manifest Finnish database (https://storage.googleapis.com/finngen-public-data r10/summary_stats/finngen_R10_C3_SCLC_EXALLC.gz), including 717 cases (584 male patients and 133 female patients) and 314,193 healthy controls of European descent. For lung adenocarcinoma, data were obtained from the C3_NSCLC_ADENO_EXALLC within the same database (https://storage.googleapis.com/finngen-public-data-r10/summary_stats/finngen_R10_C3_NSCLC_ADENO_EXALLC.gz), comprising 1,590 cases (1048 male patients and 542 female patients) and 314,193 controls of European origin. The data pertaining to lung squamous cell carcinoma were retrieved from the C3_NSCLC_SQUAM_EXALLC section (https://storage.googleapis.com/finngen-public-data-r10/summary_stats/finngen_R10_C3_NSCLC_SQUAM_EXALLC.gz), comprising 1,510 cases (1335 male patients and 175 female patients) and the same number of controls. Furthermore, the study integrated information on 41 circulating inflammatory cytokines from a comprehensive reanalysis of GWAS data related to serum cytokines 22 (https://doi.org/10.5523/bris.3g3i5smgghp0s2uvm1doflkx9x). This analysis amalgamated information from the Finnish Cardiovascular Risk in Young Adults study (n=1980; mean age: 37.4 years for men and 37.5 years for women) and the FINRISK survey (FINRISK1997: n=4608; average age: males -48.3 years; females -47.3 years / FINRISK2002: n=1705; mean age: men -60.4 years; women -60.1 years)., collectively involving 8,293 individuals of European ancestry, spanning from 1980 to 2011. Adjustments were made for age, sex, and the top ten genetic principal components to control for population structure, thereby minimizing potential confounding biases. The separation between exposure groups (inflammatory cytokines) and outcome groups (lung cancer types) in the population selection process was maintained to avoid overlap and ensure the integrity of the MR analysis.

### Statistical analysis

In this study, we primarily applied the inverse variance weighting (IVW) method for MR analysis, selected for its efficiency and statistical strength 23. It's important to note that IVW presumes all genetic variants to be valid instrumental variables, which might not always be the case in reality. To validate the causal inferences drawn, we employed additional analyses: the MR-Egger method was used to test for horizontal pleiotropy. Simple mode/Weighted mode methods helped assess the stability of our results against potential biases. Additionally, the MR-PRESSO test helped identify and remove SNPs potentially affected by confounding biases, thereby refining our dataset. A leave-one-out analysis evaluated the influence of each individual SNP on the overall results to ensure the robustness of our findings 24-27. When significant results were obtained using the IVW method (p < 0.05), conclusions were deemed reliable if the consistency of the β-value was maintained across different methods, especially in scenarios free from horizontal pleiotropy, confounding biases, or heterogeneity. The MR-Egger approach was specifically applied in cases with suspected horizontal pleiotropy but without apparent heterogeneity, whereas the weighted median and random effects IVW methods were employed in instances of heterogeneity, provided there was no polymorphism 28, 29. All data analyses were conducted using R version 4.3.2, utilizing the "TwosampleMR" and "MR-PRESSO" packages 29, 30, ensuring a comprehensive and methodical execution of MR analysis.

## Results

### Genetic predictors linked to 41 circulating inflammatory factors and diverse forms of lung cancer

To ensure a sufficient number of SNPs for the MR analysis, we established a significance threshold of *p* < 5 × 10^-6^ for SNPs linked to each circulating inflammatory factor and outcomes related to SCLC, lung adenocarcinoma, and lung squamous cell carcinoma. Following the exclusion of weak instrumental variables and SNPs associated with confounders related to outcomes, the analysis revealed 456 SNPs associated with the 41 inflammatory cytokines. Specifically, there were 10 SNPs associated with lung adenocarcinoma, 11 SNPs associated with lung squamous cell carcinoma, and 5 SNPs associated with SCLC. The subsequent screening of SNP analysis was employed in the ensuing MR process to effectively mitigate the impacts of confounding factors on the analysis. The F-statistics for the 41 inflammatory cytokine SNPs varied significantly, demonstrating a robust set of instrumental variables, with values ranging from 20.77 to 782.3. For SNPs associated with lung adenocarcinoma, F-statistics were observed to range from 21.04 to 55.18, while for lung squamous cell carcinoma, they ranged from 20.85 to 71.40. Additionally, F-statistics for SNPs associated with SCLC ranged from 20.93 to 26.34. Notably, all instrumental variables utilized in our study exhibited F-statistics greater than 10, confirming the absence of weak instrumental variable bias. Detailed information on the instrumental variables selected for this study and their corresponding F-statistics are illustrated in Supplementary Tables S1 and S2.

### Effects of 41 inflammatory cytokines on the occurrence of three types of lung cancer

**Figure 2:**
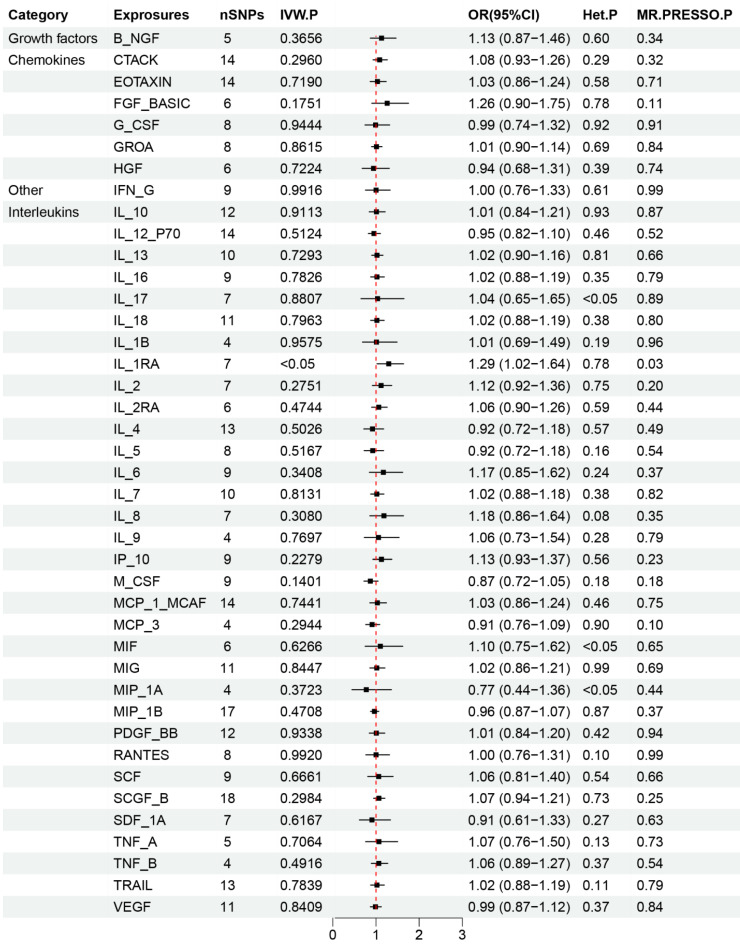
Results of Forward Mendelian randomization of inflammatory cytokines and lung adenocarcinoma.

**Figure 3:**
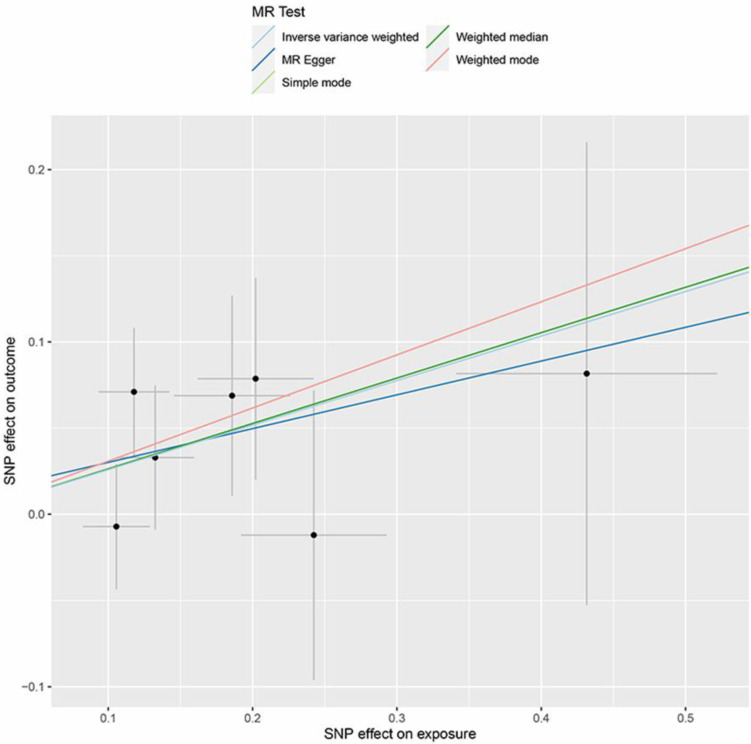
Scatter plot of the association between IL-1RA and lung adenocarcinoma. The five methods applied in the current manuscript were all depicted.

The critical outcomes of our MR analysis, illustrating the associations between 41 circulating inflammatory factors and lung adenocarcinoma, are visually detailed in Figure 2 and concisely summarized in Supplementary Table S3. In this analysis, we particularly focused on the role of inflammatory markers as predictive variables with lung adenocarcinoma as the outcome of interest. The primary findings from the Inverse Variance Weighting (IVW) method indicated a significant negative correlation between the increased genetic expression of IL_1RA and the likelihood of developing lung adenocarcinoma (OR: 1.29, 95% CI: 1.02-1.64, *p*=0.031). Additionally, MR-Egger analysis showed no indication of pleiotropy (*p*=0.85) or heterogeneity (*p*=0.67) concerning IL_1RA, reinforcing the reliability and sensitivity of these results. Moreover, the MR-PRESSO test confirmed no outliers among the IL_1RA SNPs, further solidifying the findings. To enhance the validity and sensitivity of these observations, a leave-one-out analysis was executed, yielding consistent β values compared to those derived from MR-Egger, Simple mode, Weighted median, and Weighted mode analyses, thus supporting the initial results from the IVW analysis. Figure 3 and Supplementary Figure S1 graphically delineates the causal linkage between IL_1RA levels and the incidence of lung adenocarcinoma, distinguishing this relationship from others between various inflammatory factors and the specified outcome. This set of analyses underpins the robustness and reliability of the discerned connection between IL_1RA and lung adenocarcinoma, as demonstrated by the collected data.

**Figure 4:**
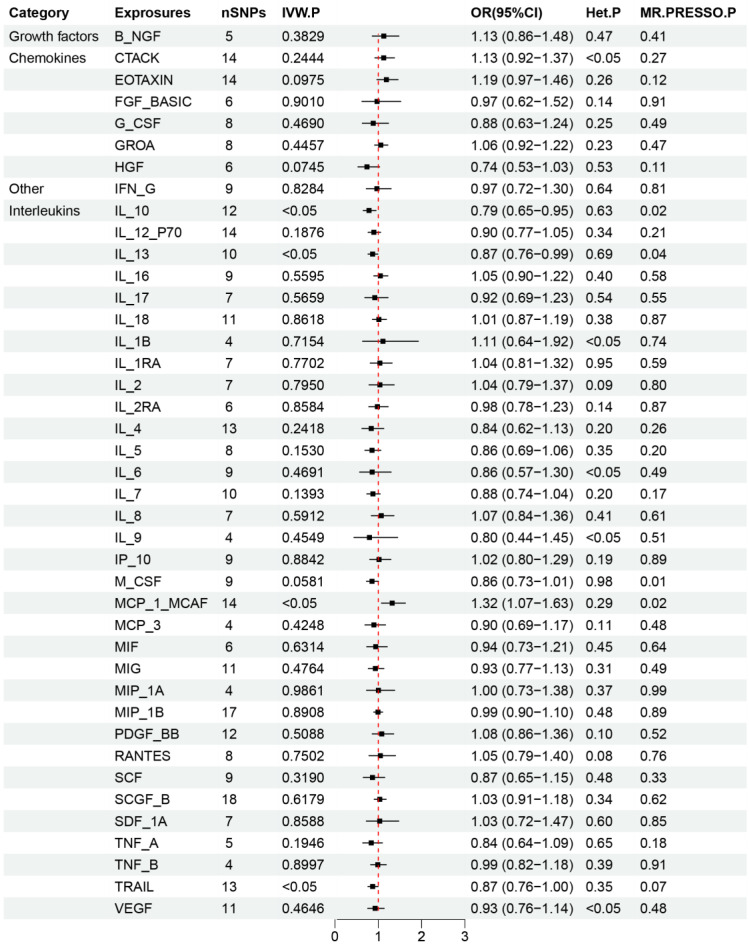
Results of Forward Mendelian randomization of inflammatory cytokines and squamous cell lung carcinoma.

**Figure 5:**
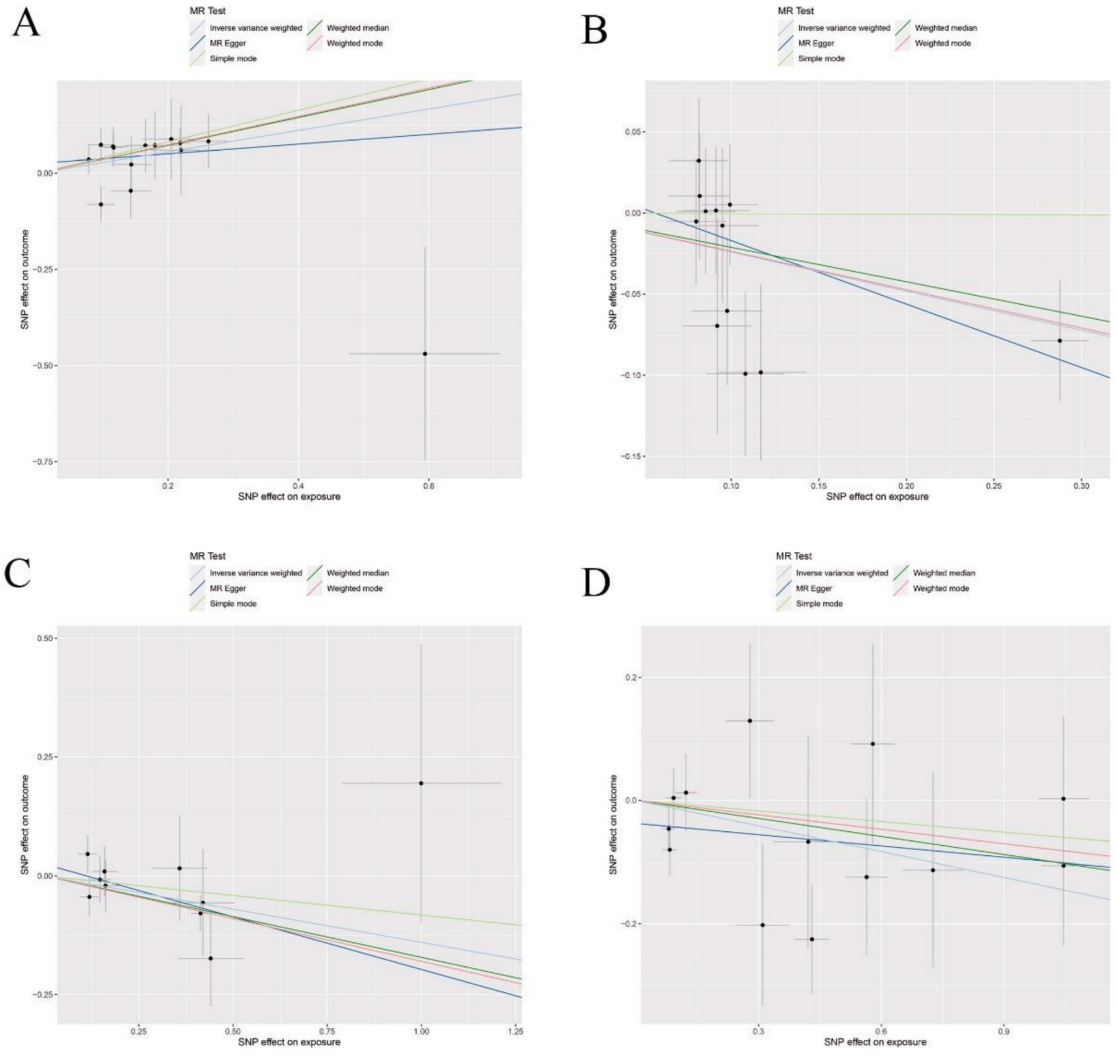
Scatter plots of Forward Mendelian randomization (MR) analyses for MCP-1, IL-10, IL-13 and TRAIL in squamous cell lung carcinoma, The five methods applied in the current manuscript were all depicted : (A) Scatter plot of the association between MCP-1and squamous cell lung carcinoma.; (B) Scatter plot of the association between IL-10 and squamous cell lung carcinoma; (C) Scatter plot of the association between IL-13 and squamous cell lung carcinoma.; (D) Scatter plot of the association between TRAIL and squamous cell lung carcinoma.

The primary results from the MR study are depicted in Figure 4 and detailed in Supplementary Table S4, where we analyzed the impact of 41 inflammatory markers on the risk of developing lung squamous cell carcinoma. The IVW analysis showed that higher levels of MCP_1_MCAF predicted by genetics were associated with a reduced risk of lung squamous cell carcinoma (OR: 0.77, 95% CI: 0.61-0.98, *p*=0.031). In contrast, elevated genetically predicted levels of IL_10, IL_13, and TRAIL were linked to an increased risk of this cancer type (IL_10: OR: 1.27, 95% CI: 1.06-1.53, *p*=0.012; IL_13: OR: 1.15, 95% CI: 1.06-1.53, *p*=0.036; TRAIL: OR: 1.15, 95% CI: 1.06-1.53, *p*=0.043). Further analyses, including MR-Egger, did not reveal any evidence of horizontal pleiotropy for these inflammatory factors (MCP_1_MCAF: *p*=0.56; IL_10: *p*=0.41; IL_13: *p*=0.47; TRAIL: *p*=0.18), nor did heterogeneity tests suggest discrepancies (MCP_1_MCAF: *p*=0.29; IL_10: *p*=0.63; IL_13: *p*=0.69; TRAIL: *p*=0.35). Additionally, the MR-PRESSO results demonstrated consistency with the identified associations of SNPs related to the investigated inflammatory factors, thus enhancing the overall robustness and sensitivity of this study. The validation of the robustness and sensitivity of our results was further confirmed through a leave-one-out analysis. Furthermore, the β values obtained from various alternative MR methods, such as MR-Egger, Simple mode, Weighted median, and Weighted mode analyses, consistently aligned with the results of the IVW analysis. These analytical layers strengthen the integrity of the conclusions. Figure 5 and Supplementary Figure S2-S4 elucidates the specific causal relationships identified between MCP_1_MCAF, IL_10, IL_13, TRAIL, and lung squamous cell carcinoma risk, distinguishing these particular cytokines from other studied factors in relation to lung cancer development.

The core findings from the MR study, as shown in Supplementary Figure S5 and detailed in Supplementary Table S5, explored the connection between 41 circulating inflammatory factors and the risk of SCLC. Our analysis revealed that there appeared to be no significant link between the evaluated circulating inflammatory cytokines and the development of SCLC. This conclusion is based on the compilation of comprehensive data, indicating that none of the studied inflammatory factors significantly contribute to the occurrence of SCLC.

### Effects of three different types of lung cancer on 41 inflammatory cytokines

The main outcomes from our MR analysis, detailed in Supplementary Figure S5 and detailed in Supplementary Table S5, explored the relationship between SCLC incidence and 41 different circulating inflammatory markers. Due to the restricted number of SNPs, we could not establish causal links for IL17, TNFB, MIP-1B, and MCP3 concerning SCLC. Consequently, our discussion is limited to the association between SCLC and the remaining 37 inflammatory cytokines. Our IVW findings suggested a positive correlation between levels of SDF_1A and B-NGF and the occurrence of SCLC (SDF_1A: OR: 1.13, 95% CI: 1.05-1.21, p=0.001; B-NGF: OR: 1.13, 95% CI: 1.01-1.27, p=0.029). No horizontal pleiotropy or heterogeneity was detected in the MR-Egger analysis for these cytokines, ensuring the robustness and sensitivity of our results (SDF_1A: p=0.78, B_NGF: p=0.23 for pleiotropy; SDF_1A: p=0.72, B_NGF: p=0.31 for heterogeneity). The MR-PRESSO test, identifying no outliers, and a leave-one-out analysis, which yielded consistent β values, further validated these findings and enhanced the sensitivity of this study.

**Figure 7:**
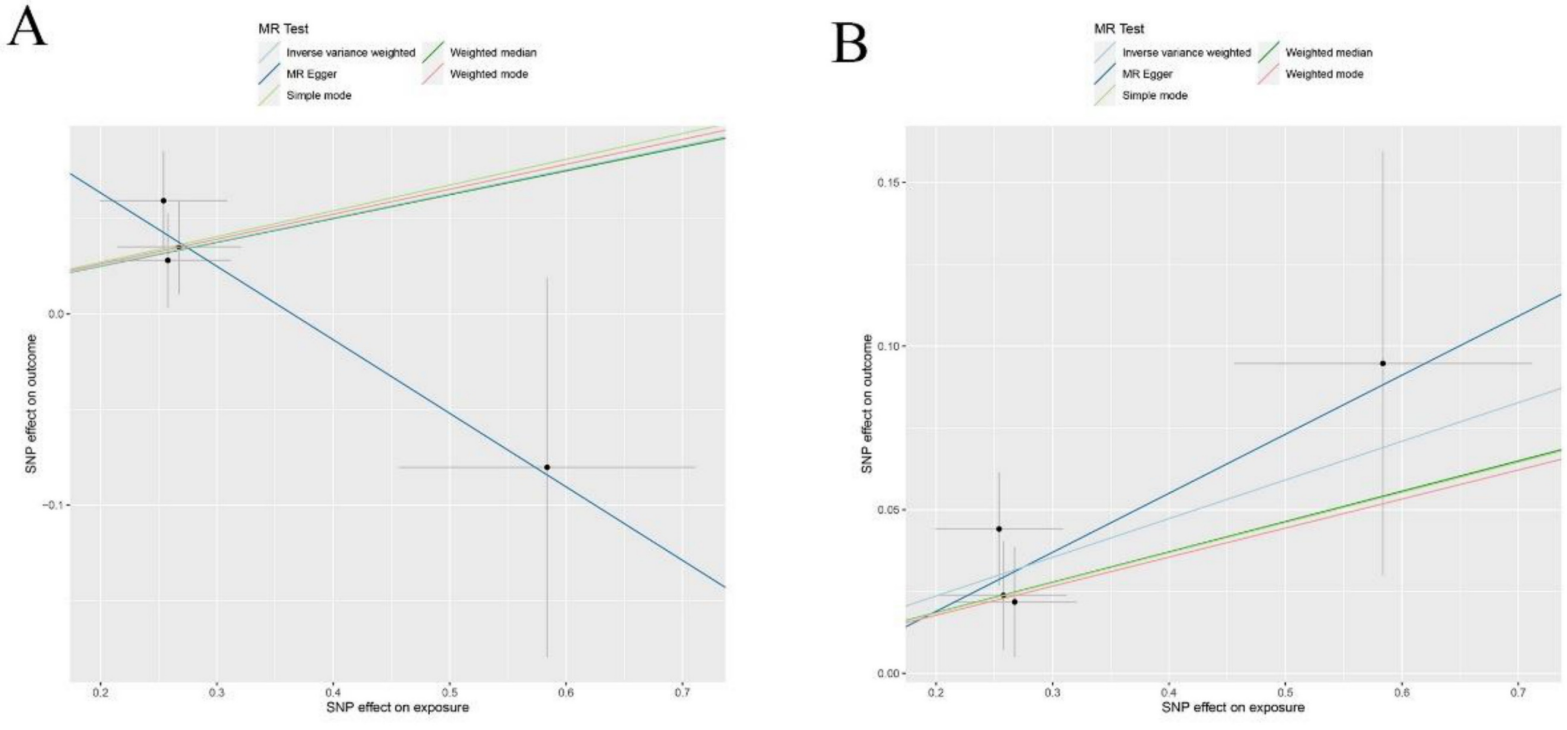
Scatter plots of Reverse Mendelian randomization (MR) analyses for B-NGF and SDF-1A in squamous cell lung carcinoma, the five methods applied in the current manuscript were all depicted: (A) Scatter plot of the association between B-NGF and squamous cell lung carcinoma; (B) Scatter plot of the association between SDF-1A and squamous cell lung carcinoma.

Figure 7 and Supplementary Figure S6-S8 provides a visual representation of the causal relationships between SCLC exposure and changes in levels of SDF_1A and B-NGF. Our analysis indicated no significant associations between SCLC and the other studied inflammatory cytokines. Additionally, as depicted in Supplementary Figure S9 and Supplementary Figure S10, alongside Supplementary Table S7 and Supplementary Table S8, no significant relationships were found between lung adenocarcinoma and lung squamous cell carcinoma with the levels of the 41 circulating inflammatory cytokines, underscoring the specificity of SCLC's link to SDF_1A and B-NGF.

## Discussion

This study aimed to determine the causal relationships between inflammatory cytokines and three specific types of lung cancer using a two-sample two-way MR analysis. We investigated the connections between 41 inflammatory biomarkers, including chemokines, interleukins, and growth factors, and the onset of lung adenocarcinoma, squamous cell carcinoma, and SCLC. Key findings demonstrated that higher gene-predicted levels of IL_1RA were associated with an increased risk of lung adenocarcinoma. In contrast, elevated gene-predicted levels of IL_10, IL_13, and TRAIL were found to reduce the risk of squamous cell carcinoma. Additionally, higher levels of MCP_1_MCAF, as predicted by genetic analysis, were positively associated with a higher risk of lung squamous cell carcinoma. However, our analysis did not link any of the examined inflammatory cytokines with SCLC outcomes. When analyzing SCLC as an exposure factor, increased levels of SDF_1A and B_NGF were observed, indicating potential pathogenic pathways. Nonetheless, no association was found between the 41 inflammatory cytokines and the outcomes of lung adenocarcinoma or squamous cell carcinoma. In summary, our findings suggest that certain biomarkers may contribute to the pathogenesis of lung adenocarcinoma and squamous cell carcinoma, while others may act as downstream factors in the progression of SCLC. Our study uniquely identified IL-1RA as a potential risk factor for lung adenocarcinoma and revealed that MCP-1, IL-10, IL-13, and TRAIL were significant factors for lung squamous cell carcinoma. Additionally, a strong correlation was observed between SDF_1A and B_NGF with SCLC. These findings offer novel clinical insights into differentiating between various subtypes of lung cancer based on inflammatory profiles, potentially informing targeted therapeutic approaches and non-invasive diagnostic strategies.

### Causal relationship between 41 circulating inflammatory factors and lung adenocarcinoma

Our study highlighted IL_1RA as a potential risk factor for lung adenocarcinoma among 41 assessed circulating inflammatory cytokines. IL-1RA, known for its role in blocking IL-1-mediated cell signaling, typically functions as a tumor suppressor but can exhibit diverse effects depending on the cancer type and pathological context 31-34. For instance, increased IL-1β levels in colon cancer have been linked to higher IL-1RA levels, impacting tumor behaviors such as invasion and apoptosis 35. In esophageal cancer, IL-1RA contributes to the inhibition of cell growth by blocking IL-1α 36 and impacts lymphangiogenesis and metastasis through the regulation of VEGF-C and MMP9 34. Conversely, in cervical and oral squamous cell carcinoma, IL-1RA levels have been associated with patient prognosis and tumor progression through various pathways 37,38. Alberto Mantovani et al. 33 suggested that IL-1RA could inhibit the "alarm" function of IL-1α, triggering protective immunity for skin cancer. While IL-1RA's role in lung cancer remains under debate, our findings suggested it may contribute to the development of lung adenocarcinoma. This might be due to the varying roles of IL-1RA in different disease stages, cell microenvironments, or its overall levels in circulation 39,40. Although previous studies like Zhao Yang et al.'s MR analysis present differing data sources and outcomes 12, the general trend aligns with our findings, indicating IL-1RA's potential involvement in lung adenocarcinoma pathogenesis. Further research is necessary to solidify these associations and to better understand the differential roles of IL-1RA in cancer. Our focus on circulating IL-1RA levels and their implications on lung adenocarcinoma provides new directions for exploring inflammatory cytokines' influence on cancer development and progression.

### Causal relationship between 41 circulating inflammatory factors and lung squamous cell carcinoma

Our results suggested that MCP_1_MCAF could act as a risk factor for lung squamous cell carcinoma. In contrast, IL_10, IL_13, and TRAIL may function as protective agents against this type of lung cancer. Interestingly, the occurrence of lung squamous cell carcinoma does not seem to significantly alter the levels of these inflammatory cytokines in the bloodstream. This distinction points towards the complex interplay between inflammation and cancer development, indicating specific cytokines might influence the pathogenesis of lung squamous cell carcinoma differently.

IL-13 is a multifunctional cytokine, mainly produced by T-helper type 2 (Th2) cells, with notable roles in inflammation and immune regulation 41,42. It shares many features with IL-4, including receptor usage and regulatory elements, signaling predominantly through the type II IL4R (combining IL4Rα and IL13Rα1) or the IL13Rα2 receptor 43-45. The impact of IL-13 on cancer is complex and not fully understood. While it is known to influence macrophage behavior, potentially supporting anti-inflammatory or tumor-friendly environments 46, its exact role can differ vastly between cancer types and stages. For instance, in certain contexts, IL-13 has been associated with promoting conditions favorable to cancer, such as through ROS-induced prooxidative environments linked to colorectal cancer progression 47-49. Conversely, other studies highlight its potential in inhibiting cancer cell adhesion and promoting tissue homeostasis, thereby acting as a deterrent to the development of cancers like those found in the skin, lungs, and intestines 50-53. In our study, IL-13 emerged as a potential protective factor against lung squamous cell carcinoma, suggesting a complex interplay in its function, highly dependent on the cancer's pathobiological context and disease stage 54.

The cytokine IL-10, produced by various immune cells and encoded by the IL-10 gene on chromosome 1q32, plays a critical role in modulating inflammatory responses 55,56. The function of IL-10 in cancer development, however, is subject to debate due to its dual effects. Some researchers, such as Ling Chen et al. 57 and Yoon Ju Jung et al. 58 have reported that IL-10 may suppress the body's anti-tumor responses, particularly in gastric cancer. Conversely, Julius Malte Vahl et al. 59 and Y.W. Pan et al. 60 have found evidence suggesting IL-10 could support tumor tolerance and growth, particularly in lung cancers. Li Yang et al. 61 have also posited that IL-10 may encourage carcinogenesis, specifically in NSCLC. Yet, IL-10 is not universally detrimental in cancer contexts. For example, Daniel F. Zegarra Ruiz et al. found that IL-10 might mitigate colitis and inhibit colitis-associated colorectal cancer development 62, while Meng Qiao et al. identified IL-10's role in enhancing immunotherapy effectiveness in specific lung cancer types 63. Narmeen Ahmad et al.'s study 64 associated IL-10 with a favorable prognosis in early invasive breast cancer. This dichotomy stems from IL-10's diverse roles in different tumor environments 65,66. IL-10 may either support or inhibit tumor development, depending on factors such as the type of cancer, the stage of disease, and the local tumor microenvironment. In certain contexts, IL-10 can inhibit macrophage activity and promote regulatory T-cell function, potentially aiding tumor escape from immune surveillance 67,68. Conversely, IL-10 can also bolster anti-tumor immune responses under certain conditions, for example, by stimulating cytotoxic CD8+ T cells and reducing inflammation 68-71. Our study suggested that IL-10 could act as a protective factor against lung squamous cell carcinoma, adding to the complex narrative surrounding this cytokine. Despite the varying perspectives, it's clear IL-10's impact on cancer is multifaceted, emphasizing the need for context-specific evaluations in understanding its role in oncogenesis and treatment.

Tumor necrosis factor-related apoptosis-inducing ligand (TRAIL/Apo2L) is part of the tumor necrosis factor superfamily and operates as a type II transmembrane protein 72,73. It is known for its unique ability to induce apoptosis selectively in cancer cells without harming normal cells by binding to death receptors DR4 and DR5 on the cancer cell surface 74,75. TRAIL also plays a role in immune-mediated tumor surveillance and suppression, primarily through actions of T cells and natural killer cells 72,76. This protein has been researched for its apoptotic effects in a variety of cancers, including those affecting the colon, lungs, bladder, breasts, kidneys, brain, prostate, and skin 77-87. In our analysis, TRAIL was identified as a protective factor specifically against lung squamous cell carcinoma, highlighting its potential as a therapeutic agent. To enhance the delivery and effectiveness of TRAIL in chemotherapy, research is currently focusing on vehicles like mesenchymal stem cells and nanoparticles 88-91. However, the challenge of TRAIL resistance in some cancer cells persists, prompting clinical trials 84 that explore overcoming this resistance through combination therapies with natural products or synthetic drugs such as disipramine, liensinine, and glipizide, aiming to sensitize these resistant tumor cells to TRAIL-induced apoptosis 87, 92-94.

Monocyte chemoattractant protein-1 (MCP-1 or CCL2) plays a critical role in immune responses and has been widely studied for its effects on monocyte attraction in vitro 95-98. This chemokine has been linked to the progression and severity of various types of cancer, including breast, lung, and esophageal cancers, as it supports tumor growth, angiogenesis, and metastasis 98-109. Our study also identified MCP-1 as a risk factor for lung squamous cell carcinoma. The CCL2/CCR2 pathway is particularly noted for its role in fostering environments conducive to cancer spread, as demonstrated by Yuqing Dong et al., who found its involvement in lymph node metastasis in tongue cancer through the activation of the RhoA and Rac1 pathways 103. MCP-1 operates by binding to its specific receptor CCR2, initiating signaling pathways that promote cancer cell proliferation and suppress immune responses 110-112. Current research efforts are focused on disrupting this pathway as a therapeutic strategy 113. Clinical trials have explored the use of anti-MCP-1 antibodies or CCR2 antagonists, showing promising results in treating breast and prostate cancers 105,114,115. These studies underscore the potential of targeting the MCP-1 signaling pathway in cancer therapy, offering new directions in the fight against tumor progression and metastasis.

### Causal relationship between 41 circulating inflammatory factors and SCLC

Our analysis revealed no significant link between the 41 circulating inflammatory markers and the development of SCLC. However, an interesting observation emerged regarding SDF_1A and B_NGF levels: their elevation appeared to be associated with the occurrence of SCLC. This suggests that while these inflammatory cytokines may not initiate SCLC, they could play a role in its progression or be indicative of the disease's presence. This insight points to the potential utility of SDF_1A and B_NGF as biomarkers in the pathogenesis of SCLC, highlighting the need for further investigation into their roles and mechanisms within this specific cancer type.

Stromal-derived factor-1 (SDF-1), also recognized as CXCL12, is a chemokine found in multiple tissues such as the brain, heart, and lungs, among others. It significantly influences the immune system through its chemotactic effects on lymphocytes 116. CXCL12 binds to its primary receptor, CXCR4 117, and recent research has also identified CXCR7 as a second receptor, highlighting its importance in cellular signaling and function regulation 118. Extracellular CXCL12 binds to either CXCR4 or CXCR7 on cell surfaces to regulate cellular functions by activating multiple signal transduction pathways 119. The interaction between CXCL12 and its receptors has been implicated in the progression and metastasis of various cancers, including ovarian, lung, breast, cervical, colon, gastric, and esophageal cancers 117, 119-126. Our research supported these findings by demonstrating an increase in circulating levels of SDF-1 in cases of SCLC, further emphasizing CXCL12's role in cancer biology. There is growing interest in targeting the CXCL12-CXCR4 and CXCL12-CXCR7 pathways for cancer therapy. Treatments developed so far, such as bicyclomycin (AMD3100), T22 peptide analogues, and dual inhibitors like GMI1359, focus on blocking these interactions 127-131. Evidence suggests that inhibiting these pathways can significantly reduce cancer cell proliferation and metastasis, offering promising prospects for future therapeutic strategies against a range of cancers 68,121,132. This approach could provide new avenues for cancer treatment, particularly for those cancers where SDF-1 levels are elevated.

B-NGF, an essential member of the nerve growth factor (NGF) family, shares similar biological activities with NGF. NGF is a critical neurotrophic factor that ensures the survival and differentiation of neuronal cells and is vital for the development and upkeep of the nervous system 133. Research indicates that NGF, along with B-NGF, is involved in angiogenesis and can facilitate cancer progression 134-137. In various types of cancers, including breast, prostate, liver, and oral cancers, B-NGF has been associated with promoting tumor initiation, progression, and metastasis 133, 136-142. Our findings align with these studies, highlighting an increase in B-NGF levels in cases of SCLC, suggesting B-NGF might enhance tumor growth and the spread of SCLC. The involvement of B-NGF in the expansion and metastasis of SCLC suggests a potential positive feedback loop that could exacerbate the disease's progression. However, the specific mechanisms and potential therapeutic applications of B-NGF in cancer, especially SCLC, remain under-explored and merit further investigation to fully understand its role and impact.

### Strengths and limitations

In a recent comprehensive study, we expanded upon previous single-item MR findings that linked CCL27/CTACK positively with lung cancer incidence in non-smokers, and IL-18 negatively with lung cancer and lung adenocarcinoma rates. For the first time, we conducted a two-way MR analysis focusing on 41 inflammatory cytokines across three distinct lung cancer types: lung adenocarcinoma, lung squamous cell carcinoma, and SCLC, utilizing data from the 10th-generation Finnish database. Our results demonstrated distinct associations between different inflammatory cytokines and the three types of lung cancer, with no overlapping cytokines among the different cancer types. This suggests that the specific inflammatory markers linked with each cancer type could serve as indirect indicators for classifying lung cancer pathologies. Consequently, this finding could potentially streamline the approach to lung cancer treatment, offering a method for guiding therapeutic decisions without necessitating invasive pathological biopsies.

Our study faces several limitations that should be considered: firstly, the data used were extracted from four large-scale GWAS, but specific demographic details and clinical records of the participants were unavailable. This absence restricts our ability to conduct subgroup analyses, which could provide more detailed insights. Secondly, we set the significance threshold for the GWAS data for exposure variables at *p* < 5 × 10^-6^, less stringent than the typical *p* < 5 × 10^-8^. This approach was necessary due to the limited number of SNPs meeting the more stringent threshold, which could otherwise hamper our ability to conduct comprehensive MR analyses. Thirdly, while results from alternative MR estimation methods such as MR-Egger, weighted mean, and simple models were not significant, the IVW method's higher statistical power and the consistency in the direction of β values allow us to still consider these findings relevant. Fourthly, the GWAS data were exclusively derived from European populations, limiting the generalizability of our findings across different racial and ethnic groups. It is essential to interpret these results with caution when applying them to non-European populations. Further research, incorporating diverse demographic groups and more detailed patient information, is needed to validate these findings and assess their applicability in clinical settings.

## Conclusion

Gene analysis has shown that increased levels of IL_1RA are associated with a higher risk of developing lung adenocarcinoma. Conversely, genetic predictions of elevated IL_10, IL_13, and TRAIL levels appear to act as protective factors against lung squamous cell carcinoma. Furthermore, a rise in MCP_1_MCAF, as suggested by gene data, indicates a heightened risk for lung squamous cell carcinoma. Currently, there is no established link between circulating inflammatory cytokines and the prognosis of SCLC.

SCLC patients exhibit elevated levels of SDF_1A and B_NGF. Conversely, there is no indication that alterations in the levels of the 41 examined circulating inflammatory cytokines influence lung adenocarcinoma and lung squamous cell carcinoma. This differentiation implies that various types of lung cancer might possess unique inflammatory profiles, offering potential clinical insights into utilizing changes in inflammatory factor levels for future lung cancer diagnostics. This approach could be particularly valuable for distinguishing between different pathological types of lung cancer and tailoring non-invasive diagnostic and treatment strategies accordingly.

## Supplementary Material

Supplementary figures and tables.

## Figures and Tables

**Figure 6:**
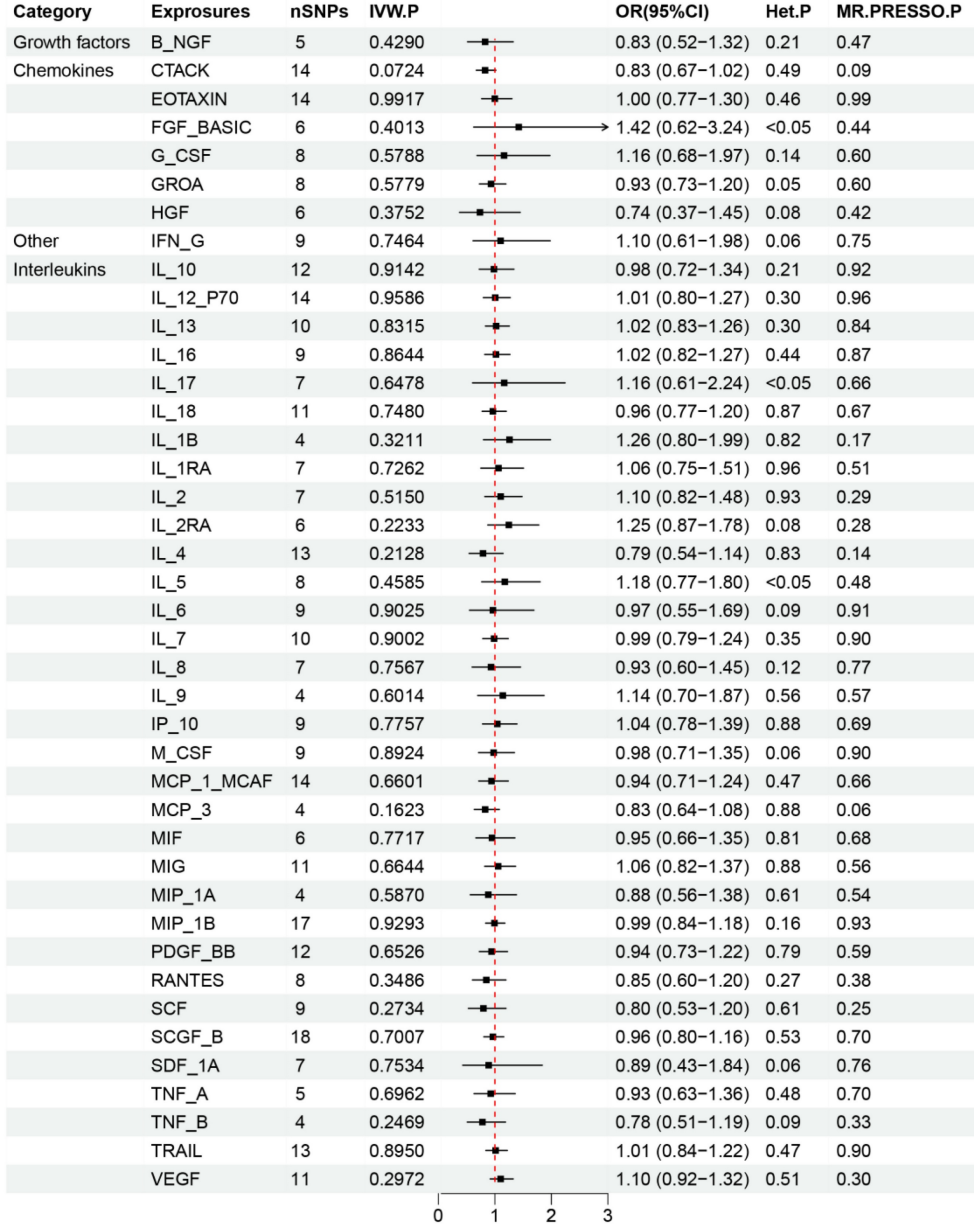
Results of Forward Mendelian randomization of inflammatory cytokines and small cell lung cancer.
