# Supplementary material for: Bidirectional Mendelian Randomization of Causal Relationship between Inflammatory Cytokines and Different Pathological Types of Lung Cancer
Source: J Cancer. 2024 Jul 16;15(15):4969–84. doi: 10.7150/jca.98301 (PMC11310887; doi:10.7150/jca.98301)
Supplement: Supplementary file 1 — Supplementary figures and tables. [file jcav15p4969s1.pdf]

Table S1 Summary of F-value of SNPs of 41 inflammatory cytokines			
Number	inflammatory cytokines	SNPs	F value
1	B-NGF	rs71641308	21.05394468
		rs28637706	35.43052959
		rs7970581	23.50932213
		rs79343884	21.5190909
		rs73472576	20.83427642
2	CTACK	rs7333764	22.61049094
		rs118084576	21.41480155
		rs62578137	21.00083583
		rs135564	39.19343126
		rs76395525	23.0647377
		rs116303454	21.46751433
		rs75077631	21.53448229
		rs57338032	20.84115386
		rs60247384	21.18601587
		rs72729450	21.91680939
		rs2070074	139.8878902
		rs141331414	22.681951
		rs57789542	21.45775354
		rs55764737	31.44486078
3	EOTAXIN	rs12075	119.1327985
		rs79722574	23.13596322
		rs75426604	22.19131072
		rs187131	24.95439978
		rs11087905	25.74394351
		rs80341932	24.50622607
		rs59808887	22.74413365
		rs2027855	21.03011373
		rs2228467	203.7234533
		rs5754733	24.2948048
		rs112347425	33.38852498
		rs745331	21.75479531
		rs2211994	24.48813453
		rs2024050	29.48273935
		rs147287945	23.32968796
		rs9317045	24.65614868
4	FGF-BASIC	rs13412535	25.39672283
		rs75168112	22.89065389
		rs61990749	24.2967822
		rs9903590	23.0123817
		rs116745220	21.7532643
		rs145577605	22.88583779
		rs78873483	20.79072111
5	G-CSF	rs117261691	20.93806517
		rs115256310	24.94224013
		rs74148555	25.07343381
		rs11903143	25.79990619
		rs586313	22.29096635
		rs2324653	25.74914397

		rs145756094	24.50789815
		rs2671444	21.84730988
		rs76287671	22.36877142
		rs77318030	22.61803121
6	GROA	rs12075	248.8571839
		rs1113500	22.85359636
		rs76390238	21.17390998
		rs185768063	28.21371586
		rs140734053	22.51444502
		rs62024303	20.82884729
		rs78653452	22.48738575
		rs118158560	21.73923431
		rs508977	189.1282386
		rs188345231	21.81961765
		rs114991247	21.81961765
7	HGF	rs1617833	21.90884386
		rs13412535	23.9720514
		rs180840563	23.61953979
		rs57146176	22.51144772
		rs4245058	21.9797747
		rs5745687	55.4228333
		rs2003620	21.85566015
		rs11060254	21.23256608
		rs3748034	43.05255388
		rs3094100	21.78996751
8	IFN-G	rs10481651	22.27489412
		rs112058662	23.53847068
		rs2073438	23.94130356
		rs11843756	21.47092273
		rs73479333	21.88894683
		rs78296352	27.66045536
		rs74148555	23.97835864
		rs12420286	22.21605764
		rs7090111	23.7448103
		rs2188420	24.99354422
		rs115729819	23.85915313
		rs113600793	25.42655913
9	IL-1B	rs61335305	21.78822809
		rs62015704	23.03655413
		rs143319329	21.9356097
		rs115242021	25.53017837
10	IL-1RA	rs187166731	23.11893754
		rs61335305	22.77133292
		rs35803309	25.2606333
		rs6699436	21.13938764
		rs11869294	23.64397273
		rs4441609	20.88659233
		rs56134659	22.07002372
		rs1054402	24.24883677
		rs139005642	22.65355055

		rs11627423	22.91838867
		rs147747784	22.55652451
11	IL-2	rs13412535	27.61819293
		rs61335305	23.62552592
		rs7615304	22.51011506
		rs16836080	20.93769294
		rs170117	22.24288719
		rs2690020	22.32737672
		rs62124990	22.14052379
		rs4634519	21.70741148
12	IL-2RA	rs115360066	22.18040218
		rs4733117	24.4400339
		rs12722497	170.0431096
		rs759244	21.11771498
		rs28441585	21.91547341
		rs185231391	22.57010899
		rs11241559	22.04965053
		rs117244812	23.16017669
		rs12799226	21.50863805
13	IL-4	rs17713451	24.79590801
		rs79597994	21.2156501
		rs73023729	24.20586363
		rs10512267	26.51600575
		rs2073438	21.41698279
		rs6969391	21.34362581
		rs2849346	21.7534839
		rs9941733	25.47641707
		rs9508291	22.01639238
		rs6765768	22.71364531
		rs117146485	20.87613122
		rs12238729	23.11643197
		rs116705532	22.84436904
		rs13106889	28.02637882
		rs7613691	21.87842355
14	IL-5	rs6737109	21.27483457
		rs148634917	22.60823615
		rs7578892	22.61655794
		rs9472168	38.38802396
		rs73040130	27.32189221
		rs28793375	21.96297799
		rs11680908	22.0531972
		rs72831687	23.35613311
		rs74811276	21.21398885
15	IL-6	rs13412535	30.70692425
		rs72831623	28.49536382
		rs4684700	21.25718053
		rs76856708	23.23311729
		rs199872130	21.35100942
		rs73273528	23.48084987
		rs113098456	20.98157092

		rs10982213	23.2640076
		rs1333040	22.63270095
		rs2404476	22.1328535
		rs75101555	21.53815214
16	IL-7	rs62006410	24.39336849
		rs115215018	20.92477696
		rs10196226	22.10880054
		rs17091524	25.15316973
		rs218247	22.19270914
		rs6921438	169.5363452
		rs117509142	22.0524712
		rs141425475	22.2287925
		rs78346957	21.10396477
		rs28793375	20.8423479
		rs77981494	24.28940097
		rs1958987	22.97559103
		rs147747784	27.96337727
17	IL-8	rs141926526	22.60795098
		rs12075	23.85087629
		rs200282774	22.97412841
		rs116726256	21.10302506
		rs12438669	21.98822436
		rs2673604	21.57013531
		rs3786107	22.68318452
		rs75840288	20.88972883
18	IL-9	rs4880409	24.59706709
		rs117807175	22.30621027
		rs41294750	21.85896323
		rs76963786	26.37122348
		rs73443903	22.07797169
		rs3736858	21.54210688
19	IL-10	rs41282660	21.17625321
		rs6085948	23.38696796
		rs3002131	20.97801343
		rs383684	21.80372694
		rs339203	22.07763131
		rs282258	37.56263067
		rs6921438	300.0883389
		rs2375980	26.15800009
		rs10493718	23.70461855
		rs3025021	22.14244769
		rs2086656	22.13959456
		rs10457128	24.64599635
		rs6799107	21.26181338
		rs7088799	24.09830926
		rs10888839	23.14934831
		rs1530455	22.20325979
		rs111913416	24.18640743
20	IL-12-P70	rs2123852	21.31753502
		rs782107	24.04192223

		rs72831623	27.62026501
		rs6993770	23.83774547
		rs282258	21.65307395
		rs6921438	559.1879504
		rs34291323	23.20750038
		rs71361173	21.55093695
		rs199840084	22.06820189
		rs2375980	35.84059101
		rs35473497	20.90114545
		rs273702	22.11950623
		rs13209117	27.81042595
		rs7088799	35.69389209
		rs41282644	21.37403558
		rs6532374	20.8871545
		rs34467391	41.70346299
21	IL-13	rs7073807	20.87896182
		rs138854806	25.09342157
		rs77955971	25.77520399
		rs12623722	21.3922198
		rs6921438	292.3603752
		rs76339001	24.3695248
		rs10995615	21.75654809
		rs27949	20.92812985
		rs6799107	24.22320659
		rs117795020	25.04196171
		rs75383097	21.4106188
		rs139083458	22.4263748
		rs147747784	23.25349581
22	IL-16	rs1801020	38.31762413
		rs116135478	25.66807914
		rs144691581	26.45695508
		rs117916513	23.02105093
		rs9706053	22.59062762
		rs117217798	21.99212902
		rs4253283	33.53177085
		rs4778636	133.6750874
		rs1255143	33.10336013
23	IL-17	rs17282552	25.26722077
		rs12735700	20.9496914
		rs78629931	25.0998356
		rs78296352	20.89867735
		rs61990749	24.72887899
		rs3804753	32.2623423
		rs149738638	21.23106573
		rs117556572	22.94315447
24	IL-18	rs12420140	90.16450936
		rs117266781	24.09662031
		rs4885797	21.07899956
		rs78716465	21.82549894
		rs17229943	44.1137792

		rs7444013	30.99227862
		rs143370787	27.2620356
		rs6517970	21.13371586
		rs385076	100.1074989
		rs1979967	24.11736945
		rs10414578	27.40403351
		rs78623212	24.64171778
		rs116383510	26.4513402
		rs4482818	25.73221616
		rs610473	27.69948592
25	IP-10	rs397816	23.83151331
		rs75970138	21.81708615
		rs6707974	21.80300482
		rs9450351	29.49487147
		rs79848609	22.06935696
		rs34383175	23.94163884
		rs143799975	21.23967378
		rs4862111	20.8538044
		rs7645625	22.34966818
		rs113831257	32.2117818
		rs8112909	21.89189711
26	M-CSF	rs9387100	21.65327982
		rs78296352	22.09770621
		rs34089869	22.53416357
		rs62294910	25.52102458
		rs56367447	30.98328962
		rs117867915	22.70060204
		rs116274860	22.553989
		rs12962919	21.05387673
		rs4269021	23.78527251
		rs11963606	20.9158207
		rs72723242	21.03455009
27	MCP_1_MCAF	rs10744620	23.64653846
		rs112313229	28.02894172
		rs12075	201.4441576
		rs7517040	26.05745937
		rs7033586	22.18745059
		rs111995966	21.35183765
		rs2228467	81.60229808
		rs138591554	94.65608414
		rs7197349	22.21262178
		rs12073356	21.45266717
		rs56212190	23.38149356
		rs146522229	26.18765785
		rs12493471	51.61450114
		rs143815843	21.00703085
		rs9317045	24.23409444
28	MCP_3	rs2838065	21.24821517
		rs28394764	21.64619403
		rs3129806	20.76669299

		rs10892381	26.38844474
		rs114363841	20.89123654
		rs62492260	23.45792105
		rs117286643	22.08928419
29	MIF	rs2330634	38.67748456
		rs2294689	21.72213525
		rs11551183	21.25224158
		rs113218956	21.93650023
		rs141009259	23.22149771
		rs12594190	24.64883752
		rs3814097	21.45685501
		rs200240331	25.97906502
		rs35890933	21.0725154
		rs13142904	27.56541915
30	MIG	rs11177248	22.39052065
		rs139010077	21.14083591
		rs6679677	24.77307927
		rs191555775	30.58165008
		rs816960	23.72267039
		rs112861654	25.88588474
		rs62562991	24.54399239
		rs13143163	22.07167987
		rs1796085	29.46906493
		rs111607343	21.87458868
		rs77086208	22.18928258
		rs55876513	41.56493045
		rs117831247	25.97246534
31	MIP_1A	rs184154340	22.25110123
		rs6900267	23.02705981
		rs12690897	21.82538303
		rs57786342	24.11086236
		rs60198979	22.39879314
		rs116615337	21.38692565
32	MIP_1B	rs113877493	782.2635389
		rs76582507	23.23648876
		rs2411161	22.17487592
		rs6908843	22.75060333
		rs116237296	22.45282839
		rs9793308	22.62417879
		rs281748	21.55479804
		rs72799710	22.83143555
		rs117657747	21.25895334
		rs113010081	603.6391313
		rs55730069	21.74571669
		rs76356863	26.84052298
		rs5743614	23.09239294
		rs1564708	82.33332327
		rs141102180	70.7659502
		rs3760440	58.76359224
		rs79068918	97.33732301

		rs17138331	23.623783
		rs1437220	20.8059525
		rs117453826	99.87439323
		rs72791296	25.72872618
		rs76776296	27.38933457
33	PDGF_BB	rs13412535	240.1923654
		rs73162807	21.48057868
		rs4965869	103.6547774
		rs11766649	21.173699
		rs35859699	21.14614404
		rs116445074	23.88255006
		rs9941733	26.3327332
		rs13037046	21.1727874
		rs72777070	27.45101307
		rs55680718	30.76114858
		rs2324229	30.14037009
		rs12289510	23.86801101
		rs9936075	22.1366206
		rs11247305	21.47450691
34	RANTES	rs147509526	24.74846445
		rs62438851	21.24135605
		rs9675798	21.88361727
		rs72793342	24.01843481
		rs112072646	23.99495709
		rs28695841	26.73837292
		rs75077631	22.09429335
		rs7170339	22.43405301
		rs7000423	27.17303215
		rs74472919	34.92756664
35	SCF	rs13412535	24.99400048
		rs113127926	22.29654125
		rs1568119	27.73049941
		rs72678285	21.13103838
		rs78666213	24.56046143
		rs78369473	21.72117089
		rs4841899	31.68043992
		rs1557570	48.08145255
		rs80271436	24.43939435
		rs635634	29.35686869
		rs7039247	22.10708007
36	SCGF_B	rs17876031	34.67067222
		rs77954165	21.90461243
		rs12480722	21.9426011
		rs181218758	41.00361644
		rs78217154	20.95045432
		rs116924815	67.26169669
		rs13287050	21.15561973
		rs4656185	68.51373136
		rs149009264	21.33574039
		rs143829871	21.85968985

		rs7815967	21.15501265
		rs264157	21.43372013
		rs1149926	21.30359273
		rs151194174	23.22380377
		rs117716477	96.22172903
		rs112346514	21.50589177
		rs150733161	22.00269627
		rs139413256	23.10972696
		rs34911860	21.78187932
37	SDF_1A	rs78037609	22.02240723
		rs149893336	20.83955385
		rs78883416	22.8972104
		rs3988298	22.53888712
		rs62194947	21.20434069
		rs10474392	27.83788654
		rs1600396	20.91182777
		rs12141941	22.42926123
		rs10013755	27.17958455
38	TNF_A	rs10834997	23.0718041
		rs79105320	22.40662385
		rs111332265	24.35915212
		rs7256693	21.17087742
		rs115669577	24.19017276
39	TNF_B	rs78296352	77.43377481
		rs75240021	23.10256968
		rs10925040	21.80010852
		rs753274	21.70799644
		rs7629875	24.59527507
40	TRAIL	rs17434886	21.27520029
		rs28521641	247.6661772
		rs747324	21.52853799
		rs138987090	94.0334745
		rs74778900	118.9084
		rs79287178	104.9881764
		rs72899452	21.4555429
		rs13278062	25.95823244
		rs28431810	23.27879609
		rs550057	21.46073356
		rs193112415	284.3427884
		rs57396456	119.4833097
		rs148051545	24.94650912
		rs62093514	362.852471
		rs75928541	22.18492414
		rs73039026	23.87144776
41	VEGF	rs10411345	22.7964477
		rs12456390	20.87753647
		rs8045833	23.82254985
		rs7030781	66.51768925
		rs6921438	781.8517602
		rs10967186	28.28946145

		rs4082730	21.20934617
		rs10761739	43.41064134
		rs13209117	39.23926431
		rs73418461	22.98201624
		rs73872715	21.89397865
		rs3108686	21.90289909
		rs143479231	28.87431627
		rs34467391	56.2342921

Table S2 Summary of F-values of SNPS for three different pathologic types of lung cancer			
Number	Pathological classification of lung cancer	SNPs	F value
1	Lung adenocarcinoma	rs34877707	21.28056531
		rs12518326	24.02997265
		rs112333466	22.46323896
		rs4865544	21.04460675
		rs148311882	21.26287681
		rs12253830	21.64523359
		rs76566554	22.30234281
		rs56077333	55.17755271
		rs7204446	22.04155053
		rs35922794	22.7988881
2	squamous cell lung carcinoma	rs17431476	25.75905351
		rs72805822	21.48502227
		rs16878358	20.86729751
		rs2939230	24.12332949
		rs11571815	23.00706669
		rs8040868	71.40423215
		rs150070907	20.85141392
		rs2848986	22.38567697
		rs185064152	21.06988418
		rs75602421	21.24148847
		rs79378907	32.87975251
3	small cell lung cancer	rs2749722	21.55273213
		rs4852278	22.51426355
		rs75760665	20.93196348
		rs12914385	25.39623084
		rs8106146	26.34296251

TableS3 Summary of results of positive Mendelian randomization of inflammatory cytokines and lung adenocarcinoma						
**Inflammatory cytokines**	**nSNP**	**IVW-OR**	**IVW-pval**	**IVW_heterogeneity-Q_pval**	**Pleiotropy-pval**	**Presso-pval**
B_NGF	5	1.127362	0.382917956	0.47222678	0.273392502	0.406007298
CTACK	14	1.125578	0.244414799	0.023863406	0.930534521	0.265333564
EOTAXIN	14	1.187901	0.097544172	0.263310791	0.136286822	0.121467566
FGF_BASIC	6	0.972101	0.901021929	0.136852951	0.579937573	0.905867281
G_CSF	8	0.883311	0.468992663	0.247776787	0.666909238	0.492477875
GROA	8	1.057533	0.445699321	0.225667223	0.594355425	0.470611381
HGF	6	0.739214	0.074508297	0.533379355	0.708447268	0.106385368
IFN_G	9	0.968237	0.82844492	0.639241151	0.870877427	0.809824725
**IL_10**	**12**	**0.785934**	**0.011614251**	**0.63114679**	**0.40926729**	**0.017100284**
IL_12_P70	14	0.898007	0.187613153	0.338876279	0.141636033	0.210363127
**IL_13**	**10**	**0.869361**	**0.036281746**	**0.688083482**	**0.471087289**	**0.036040267**
IL_16	9	1.047168	0.55948239	0.39532542	0.466781218	0.57556272
IL_17	7	0.919323	0.565900146	0.541422819	0.866116981	0.55330859
IL_18	11	1.014192	0.861750225	0.380643329	0.392477809	0.865225244
IL_1B	4	1.10813	0.71541422	0.021726593	0.712273822	0.739598949
IL_1RA	7	1.036692	0.770242228	0.951547662	0.697039657	0.593789966
IL_2	7	1.037267	0.795022662	0.088768349	0.979360589	0.803707379
IL_2RA	6	0.97966	0.858423423	0.14296813	0.377697523	0.865425763
IL_4	13	0.837364	0.241804888	0.195740182	0.041567347	0.264525368
IL_5	8	0.855728	0.153015064	0.349666169	0.097287044	0.196082598
IL_6	9	0.858891	0.469089997	0.049825908	0.279691967	0.489716237
IL_7	10	0.876687	0.139312666	0.197838814	0.067277366	0.173434882
IL_8	7	1.068704	0.591236992	0.406712491	0.168425746	0.610558044
IL_9	4	0.796132	0.454907436	0.028260705	0.783718154	0.509145267
IP_10	9	1.017804	0.884217192	0.185880454	0.411400209	0.887819556
M_CSF	9	0.855364	0.058059233	0.977635357	0.520455309	0.006085476
**MCP_1_MCAF**	**14**	**1.321174**	**0.008493442**	**0.293945904**	**0.560451647**	**0.020716508**
MCP_3	4	0.899355	0.424760301	0.108157731	0.417303342	0.483103514
MIF	6	0.940935	0.631382343	0.45489025	0.989591585	0.641324609
MIG	11	0.93275	0.476399234	0.306434941	0.213290163	0.492678282
MIP_1A	4	1.002833	0.986099375	0.374344201	0.68707855	0.987193323
MIP_1B	17	0.992827	0.890839239	0.481533558	0.482075708	0.891176137
PDGF_BB	12	1.079815	0.508755137	0.100816172	0.179170195	0.522353054
RANTES	8	1.047861	0.75024394	0.078090314	0.319800432	0.759530635
SCF	9	0.866222	0.318965016	0.484181248	0.5183515	0.333342185
SCGF_B	18	1.034453	0.617929589	0.336366984	0.10081913	0.624321899
SDF_1A	7	1.032707	0.858785714	0.599204556	0.650896424	0.845300085
TNF_A	5	0.837675	0.194621404	0.645145394	0.667464481	0.176025482
TNF_B	4	0.988427	0.89971417	0.387535207	0.504667472	0.907685657
**TRAIL**	**13**	**0.870554**	**0.043499189**	**0.347791334**	**0.175739513**	**0.066414966**
VEGF	11	0.926899	0.464594873	0.004433719	0.123904804	0.481379666

Table S4 Summary of results of positive Mendelian randomization of inflammatory cytokines and squamous cell lung carcinoma						
**Inflammatory cytokines**	**nSNP**	**IVW-OR**	**IVW-pval**	**IVW_heterogeneity-Q_pval**	**Pleiotropy-pval**	**Presso-pval**
B_NGF	5	1.127361556	0.382917956	0.47222678	0.273392502	0.406007298
CTACK	14	1.125578422	0.244414799	0.023863406	0.930534521	0.265333564
EOTAXIN	14	1.187901086	0.097544172	0.263310791	0.136286822	0.121467566
FGF_BASIC	6	0.972100857	0.901021929	0.136852951	0.579937573	0.905867281
G_CSF	8	0.883310551	0.468992663	0.247776787	0.666909238	0.492477875
GROA	8	1.057532557	0.445699321	0.225667223	0.594355425	0.470611381
HGF	6	0.73921392	0.074508297	0.533379355	0.708447268	0.106385368
IFN_G	9	0.968236744	0.82844492	0.639241151	0.870877427	0.809824725
**IL_10**	**12**	**0.785933828**	**0.011614251**	**0.63114679**	**0.40926729**	**0.017100284**
IL_12_P70	14	0.898007231	0.187613153	0.338876279	0.141636033	0.210363127
**IL_13**	**10**	**0.869361403**	**0.036281746**	**0.688083482**	**0.471087289**	**0.036040267**
IL_16	9	1.047168368	0.55948239	0.39532542	0.466781218	0.57556272
IL_17	7	0.919323098	0.565900146	0.541422819	0.866116981	0.55330859
IL_18	11	1.01419177	0.861750225	0.380643329	0.392477809	0.865225244
IL_1B	4	1.108129994	0.71541422	0.021726593	0.712273822	0.739598949
IL_1RA	7	1.036691811	0.770242228	0.951547662	0.697039657	0.593789966
IL_2	7	1.037267203	0.795022662	0.088768349	0.979360589	0.803707379
IL_2RA	6	0.979660261	0.858423423	0.14296813	0.377697523	0.865425763
IL_4	13	0.83736353	0.241804888	0.195740182	0.041567347	0.264525368
IL_5	8	0.855728364	0.153015064	0.349666169	0.097287044	0.196082598
IL_6	9	0.858891004	0.469089997	0.049825908	0.279691967	0.489716237
IL_7	10	0.87668724	0.139312666	0.197838814	0.067277366	0.173434882
IL_8	7	1.068703885	0.591236992	0.406712491	0.168425746	0.610558044
IL_9	4	0.796132346	0.454907436	0.028260705	0.783718154	0.509145267
IP_10	9	1.017803516	0.884217192	0.185880454	0.411400209	0.887819556
M_CSF	9	0.855363672	0.058059233	0.977635357	0.520455309	0.006085476
**MCP_1_MCAF**	**14**	**1.321174489**	**0.008493442**	**0.293945904**	**0.560451647**	**0.020716508**
MCP_3	4	0.899354936	0.424760301	0.108157731	0.417303342	0.483103514
MIF	6	0.940934677	0.631382343	0.45489025	0.989591585	0.641324609
MIG	11	0.932750031	0.476399234	0.306434941	0.213290163	0.492678282
MIP_1A	4	1.002832825	0.986099375	0.374344201	0.68707855	0.987193323
MIP_1B	17	0.992826998	0.890839239	0.481533558	0.482075708	0.891176137
PDGF_BB	12	1.07981476	0.508755137	0.100816172	0.179170195	0.522353054
RANTES	8	1.047860709	0.75024394	0.078090314	0.319800432	0.759530635
SCF	9	0.866222155	0.318965016	0.484181248	0.5183515	0.333342185
SCGF_B	18	1.034452782	0.617929589	0.336366984	0.10081913	0.624321899
SDF_1A	7	1.032706598	0.858785714	0.599204556	0.650896424	0.845300085
TNF_A	5	0.837674538	0.194621404	0.645145394	0.667464481	0.176025482
TNF_B	4	0.988426515	0.89971417	0.387535207	0.504667472	0.907685657
**TRAIL**	**13**	**0.870554239**	**0.043499189**	**0.347791334**	**0.175739513**	**0.066414966**
VEGF	11	0.926899141	0.464594873	0.004433719	0.123904804	0.481379666

Table S5 Summary of results of positive Mendelian randomization of inflammatory cytokines and small cell lung cancer						
**Inflammatory cytokines**	**nSNP**	**IVW-OR**	**IVW-pval**	**IVW_heterogeneity-Q_pval**	**Pleiotropy-pval**	**Presso-pval**
B_NGF	5	0.827093	0.429033038	0.205252413	0.575693794	0.473282347
CTACK	14	0.826576	0.072378405	0.486111606	0.719820319	0.090050368
EOTAXIN	14	0.99859	0.991668548	0.464601508	0.689260272	0.991758464
FGF_BASIC	6	1.422106	0.401287543	0.016212636	0.141235801	0.439561101
G_CSF	8	1.161926	0.578811778	0.142035405	0.529280594	0.59609684
GROA	8	0.931683	0.577893362	0.054404391	0.753788921	0.595226596
HGF	6	0.736393	0.375158956	0.075272666	0.314291961	0.415772599
IFN_G	9	1.101517	0.746387022	0.059213227	0.857308106	0.754679093
IL_10	12	0.983236	0.914151182	0.213521424	0.225043332	0.916092381
IL_12_P70	14	1.006229	0.958601981	0.302550281	0.156306517	0.959391127
IL_13	10	1.022523	0.831457376	0.302223963	0.333358092	0.836199988
IL_16	9	1.01914	0.864387873	0.439170711	0.4073549	0.868153657
IL_17	7	1.164844	0.647819376	0.022963192	0.932429443	0.663889913
IL_18	11	0.96466	0.747996152	0.872429099	0.699676099	0.667495147
IL_1B	4	1.259141	0.321112267	0.820846911	0.763792679	0.170932741
IL_1RA	7	1.064089	0.726222825	0.960891759	0.675861331	0.50705804
IL_2	7	1.102785	0.514997361	0.928684284	0.926979407	0.291223075
IL_2RA	6	1.24671	0.223347164	0.077893401	0.520735478	0.277672326
IL_4	13	0.789629	0.212771222	0.8269826	0.305350733	0.139624073
IL_5	8	1.175277	0.458520931	0.034686626	0.336256587	0.482643008
IL_6	9	0.965597	0.902528811	0.085675894	0.604857026	0.905549439
IL_7	10	0.985557	0.900191614	0.347918981	0.855340897	0.902948512
IL_8	7	0.932116	0.756716759	0.122733451	0.969153912	0.76719423
IL_9	4	1.140883	0.601371534	0.558826236	0.476158633	0.573672224
IP_10	9	1.042759	0.775737639	0.879165221	0.823902918	0.68817998
M_CSF	9	0.978215	0.892370636	0.064093694	0.525920862	0.895713142
MCP_1_MCAF	14	0.940326	0.660137923	0.474199528	0.474806348	0.663256901
MCP_3	4	0.828114	0.162317547	0.877889853	0.528239463	0.060760459
MIF	6	0.94868	0.771716002	0.8132449	0.749706983	0.683546358
MIG	11	1.058226	0.664438383	0.879693702	0.496356772	0.559655487
MIP_1A	4	0.883379	0.58704494	0.611921496	0.343582303	0.535151324
MIP_1B	17	0.99232	0.929326648	0.161401118	0.497893264	0.930427313
PDGF_BB	12	0.941721	0.652615083	0.785748723	0.834053225	0.588139536
RANTES	8	0.848929	0.34858557	0.269496429	0.464877057	0.379790125
SCF	9	0.796856	0.273407012	0.613689858	0.527827127	0.252142283
SCGF_B	18	0.964928	0.700723259	0.533087391	0.775128321	0.695731614
SDF_1A	7	0.890321	0.753409224	0.059878011	0.986907512	0.764045282
TNF_A	5	0.926376	0.696150397	0.480398627	0.805042006	0.697090775
TNF_B	4	0.779735	0.246880796	0.087024906	0.161380951	0.330695373
TRAIL	13	1.012614	0.894984513	0.472663018	0.136076166	0.895730436
VEGF	11	1.102303	0.297195393	0.507748715	0.415448166	0.304060868

Table S6 Summary of results of Reverse Mendelian randomization of inflammatory cytokines and small cell lung cancer						
**Inflammatory cytokines**	**nSNP**	**IVW-OR**	**IVW-pval**	**IVW_heterogeneity-Q_pval**	**Pleiotropy-pval**	**Presso-pval**
**B_NGF**	**4**	**1.134058**	**0.028728923**	**0.31421395**	**0.233959062**	**0.11656114**
CTACK	4	1.005801	0.911291317	0.84424951	0.577640554	0.845076
EOTAXIN	4	1.059861	0.342682202	0.025047822	0.092797864	0.412685348
FGF_BASIC	4	0.991472	0.81217164	0.868453504	0.573000865	0.660887252
G_CSF	4	1.060673	0.151454204	0.25185492	0.571489617	0.246930421
GROA	4	1.086521	0.11736564	0.530849294	0.309834173	0.165279558
HGF	4	1.069267	0.051412074	0.506173622	0.518404321	0.114221661
IFN_G	4	1.064462	0.080281871	0.944527895	0.971844555	0.016084351
IL_10	4	1.029112	0.42199734	0.705096352	0.895424522	0.324893907
IL_12_P70	4	1.027297	0.434489441	0.63189228	0.35323808	0.378247942
IL_13	4	0.931718	0.178309707	0.87311029	0.595414235	0.068667081
IL_16	4	1.075985	0.168870768	0.386488788	0.650113284	0.262593469
IL_18	4	0.999653	0.994670368	0.703919735	0.446027099	0.992829808
IL_1B	4	1.002692	0.960592931	0.872365774	0.511277636	0.925171513
IL_1RA	4	0.994577	0.916601947	0.867293708	0.876914033	0.844979257
IL_2	4	1.019925	0.781177684	0.147329339	0.915555027	0.799221508
IL_2RA	4	1.025658	0.827946906	0.001639169	0.063355352	0.841889128
IL_4	4	1.057522	0.108058305	0.901629937	0.607614582	0.035157086
IL_5	4	1.008234	0.878899737	0.944593322	0.955143917	0.69699283
IL_6	4	1.066519	0.063421386	0.480636089	0.383431761	0.133342592
IL_7	4	1.011027	0.837908006	0.79139375	0.576003607	0.751288704
IL_8	4	0.983374	0.754473336	0.373748481	0.734562321	0.77495333
IL_9	4	0.974882	0.682416231	0.229276049	0.979076429	0.709874949
IP_10	4	1.060407	0.255129222	0.923871541	0.835876376	0.064933523
M_CSF	4	1.071984	0.273377618	0.671684742	0.48935526	0.224488027
MCP_1_MCAF	4	1.009342	0.787961185	0.421888739	0.243620054	0.799131813
MIF	4	1.087971	0.110718474	0.427663674	0.812738771	0.195811466
MIG	4	1.035382	0.499827341	0.646408724	0.416570176	0.430923029
MIP_1A	4	0.969664	0.558302248	0.625184001	0.748133048	0.499566245
PDGF_BB	4	0.994829	0.88045903	0.787669754	0.61381453	0.816289691
RANTES	4	1.033943	0.533231517	0.898804377	0.794259331	0.254543392
SCF	4	1.053811	0.426831395	0.011538733	0.549036254	0.484888964
SCGF_B	4	1.072788	0.173883021	0.84347709	0.800221513	0.080857576
**SDF_1A**	**4**	**1.125439**	**0.000885717**	**0.724194801**	**0.782694133**	**0.015305508**
TNF_A	4	1.028857	0.597069234	0.381467289	0.54769577	0.633681061
TRAIL	4	1.012881	0.713263953	0.385305633	0.671404142	0.737657861
VEGF	4	1.020282	0.589511528	0.960136213	0.695895848	0.186206079

Table S7 Summary of results of Reverse Mendelian randomization of inflammatory cytokines and lung adenocarcinoma						
**Inflammatory cytokines**	**nSNP**	**IVW-OR**	**IVW-pval**	**IVW_heterogeneity-Q_pval**	**Pleiotropy-pval**	**Presso-pval**
B_NGF	6	0.981454	0.809139726	0.091348578	0.463395631	0.818732731
CTACK	6	1.070463	0.218532096	0.783684525	0.887879401	0.139259822
EOTAXIN	6	0.967093	0.366407741	0.812647436	0.379206597	0.236548401
FGF_BASIC	6	1.027571	0.51550711	0.30881159	0.359645931	0.544199291
G_CSF	6	0.981855	0.68670147	0.195905207	0.663766647	0.703370927
GROA	6	0.963845	0.51386228	0.800506319	0.799236563	0.383677603
HGF	6	0.974655	0.648502709	0.037764974	0.525081572	0.667616988
IFN_G	6	0.98002	0.656499045	0.212125354	0.143059326	0.675090968
IL_10	6	0.992924	0.852280805	0.984232627	0.704742205	0.634221113
IL_12_P70	6	0.954934	0.208991058	0.938906472	0.469128094	0.054367744
IL_13	6	1.005398	0.923440307	0.963625705	0.891966901	0.837283867
IL_16	6	1.060294	0.298786103	0.526921028	0.57436477	0.306155372
IL_18	6	0.973195	0.635257833	0.374235064	0.672331605	0.655250034
IL_1B	6	0.952917	0.421010678	0.375395717	0.351248958	0.457549151
IL_1RA	6	0.922445	0.18740261	0.294374925	0.297494135	0.244561382
IL_2	6	0.959839	0.53272215	0.238143099	0.555683589	0.560076741
IL_2RA	6	1.112412	0.122554171	0.16354936	0.344732538	0.183207834
IL_4	6	0.98307	0.645196497	0.480586406	0.606050642	0.647758001
IL_5	6	0.992673	0.898052798	0.882921917	0.587789186	0.836970421
IL_6	6	0.982269	0.628611439	0.932740008	0.755751203	0.389930368
IL_7	6	0.957686	0.463974219	0.377710969	0.429297321	0.496843406
IL_8	6	0.989896	0.87202348	0.277076791	0.748721886	0.878330048
IL_9	6	1.02366	0.672188836	0.722298549	0.250090399	0.599664291
IP_10	6	0.964835	0.572347496	0.246534614	0.747970423	0.596735082
M_CSF	6	1.028558	0.673826432	0.738009221	0.600427376	0.595069459
MCP_1_MCAF	6	1.031393	0.400909042	0.789403964	0.651150477	0.280730099
MCP_3	6	0.939409	0.532265643	0.630907074	0.711783943	0.486016634
MIF	6	0.966869	0.549249571	0.857846774	0.862365159	0.380096405
MIG	6	0.962212	0.588079598	0.134304465	0.598051406	0.611332254
MIP_1A	6	1.053952	0.349460072	0.946697247	0.497417682	0.112171109
PDGF_BB	6	0.961282	0.282125774	0.909695361	0.517658985	0.109374346
RANTES	6	0.957442	0.44679416	0.610035023	0.695725754	0.410336339
SCF	6	0.999353	0.985897972	0.801081561	0.731350079	0.980366773
SCGF_B	6	1.028932	0.791314777	0.001759925	0.997001298	0.80187592
SDF_1A	6	0.931019	0.059456631	0.916080633	0.605924027	0.017817003
TNF_A	6	0.991769	0.884412439	0.619164908	0.243607624	0.869384012
TNF_B	4	1.02052	0.874865712	0.154086908	0.321882449	0.884868127
TRAIL	6	1.005715	0.876971375	0.533090592	0.58928969	0.871179972
VEGF	6	0.997795	0.955601938	0.612043137	0.194136296	0.950057344

Table S8 Summary of results of Reverse Mendelian randomization of inflammatory cytokines and squamous cell lung carcinoma						
**Inflammatory cytokines**	**nSNP**	**IVW-OR**	**IVW-pval**	**IVW_heterogeneity-Q_pval**	**Pleiotropy-pval**	**Presso-pval**
B_NGF	8	0.993716535	0.895715707	0.314441907	0.216916955	0.899403298
CTACK	8	1.012169612	0.799425357	0.309622491	0.143005739	0.806727485
EOTAXIN	8	0.992574639	0.800546002	0.500254581	0.219674558	0.798354404
FGF_BASIC	8	0.995352421	0.87871812	0.848714779	0.678517934	0.832225824
G_CSF	8	0.982784536	0.560754227	0.502217814	0.647456859	0.559934616
GROA	8	0.942132106	0.409461069	0.010183955	0.701263046	0.43666307
HGF	8	0.969594624	0.29039912	0.740232086	0.140715859	0.221136325
IFN_G	8	0.981945932	0.546653797	0.702027821	0.568359261	0.483825131
IL_10	8	1.019482027	0.525369394	0.993239222	0.528461388	0.15159178
IL_12_P70	8	0.978307375	0.454581853	0.811085663	0.624656018	0.33929688
IL_13	8	0.984396395	0.722004986	0.779477598	0.33409543	0.652305306
IL_16	8	1.01436677	0.749629365	0.875136506	0.573138909	0.646421561
IL_18	8	1.057134857	0.204567018	0.529850852	0.498179921	0.21579927
IL_1B	8	1.01978214	0.685835274	0.352113221	0.661543881	0.697910061
IL_1RA	8	0.972290455	0.521491878	0.969762322	0.55702078	0.247427503
IL_2	8	0.966453898	0.445141009	0.596692861	0.486309505	0.418381185
IL_2RA	8	0.99317538	0.875106956	0.55347367	0.186787627	0.868722477
IL_4	8	0.978307069	0.458321199	0.760533064	0.623920881	0.368385707
IL_5	8	1.018731675	0.682453298	0.985119639	0.888419952	0.393038344
IL_6	8	0.979692262	0.486575849	0.893677871	0.495169138	0.315901763
IL_7	8	0.969578776	0.493816036	0.987209041	0.580752465	0.16243218
IL_8	8	0.954340297	0.292864672	0.871273941	0.827973809	0.160575183
IL_9	8	1.04705557	0.294305917	0.756243419	0.544082643	0.217948071
IP_10	8	0.971338264	0.504154316	0.572317827	0.989945324	0.484129343
M_CSF	8	0.968143893	0.681051671	0.032725812	0.087669215	0.693343172
MCP_1_MCAF	8	0.997707446	0.939981369	0.3720573	0.977096708	0.942087989
MCP_3	7	1.03527533	0.7173016	0.238877768	0.987665076	0.729711874
MIF	8	0.94838387	0.236033579	0.999825615	0.928422724	0.001158849
MIG	8	0.950168341	0.239332628	0.931270213	0.983094136	0.086707014
MIP_1A	8	1.016881777	0.706052267	0.648594289	0.513143426	0.671708465
PDGF_BB	8	0.968263204	0.271435035	0.927821325	0.481408159	0.107740249
RANTES	8	0.992411957	0.867049393	0.948416734	0.392151627	0.773533736
SCF	8	0.961426531	0.209336219	0.328079969	0.207119458	0.249618998
SCGF_B	8	0.95471424	0.286375793	0.485630661	0.054300221	0.304240489
SDF_1A	8	0.958635854	0.161766674	0.903650102	0.379016765	0.062255698
TNF_A	8	0.996222787	0.932601412	0.682336062	0.637464676	0.921651557
TNF_B	5	0.964080335	0.620476214	0.477949552	0.65258928	0.624546446
TRAIL	8	1.01650046	0.647247416	0.16833814	0.728292313	0.66111169
VEGF	8	1.00607851	0.847674065	0.971785234	0.644107382	0.71326158

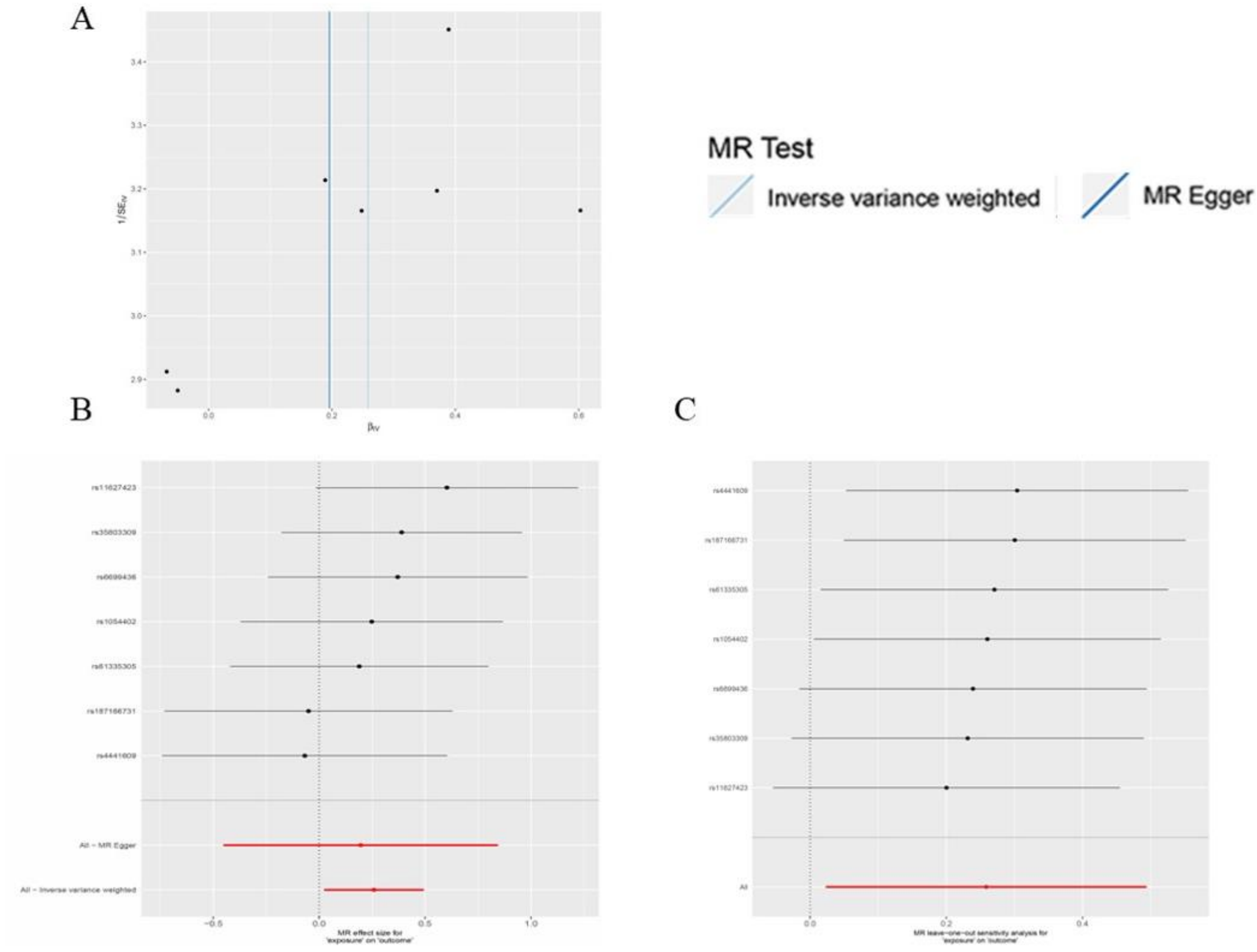

Figure S1

Funnel plots and forest plots of Forward Mendelian randomization (MR) analyses for IL-IRA in lung adenocarcinoma: (A)Funnel plot was applied to detect whether the observed association was along with obvious heterogeneity. (B) Forest plot was used to show the MR estimate and 95% CI values (gray line segment) for each SNP which also shows the IVW and MR-Egger results at the bottom. (C) Leave-one-out analyses to evaluate whether any single instrumental variable was driving the causal effect.

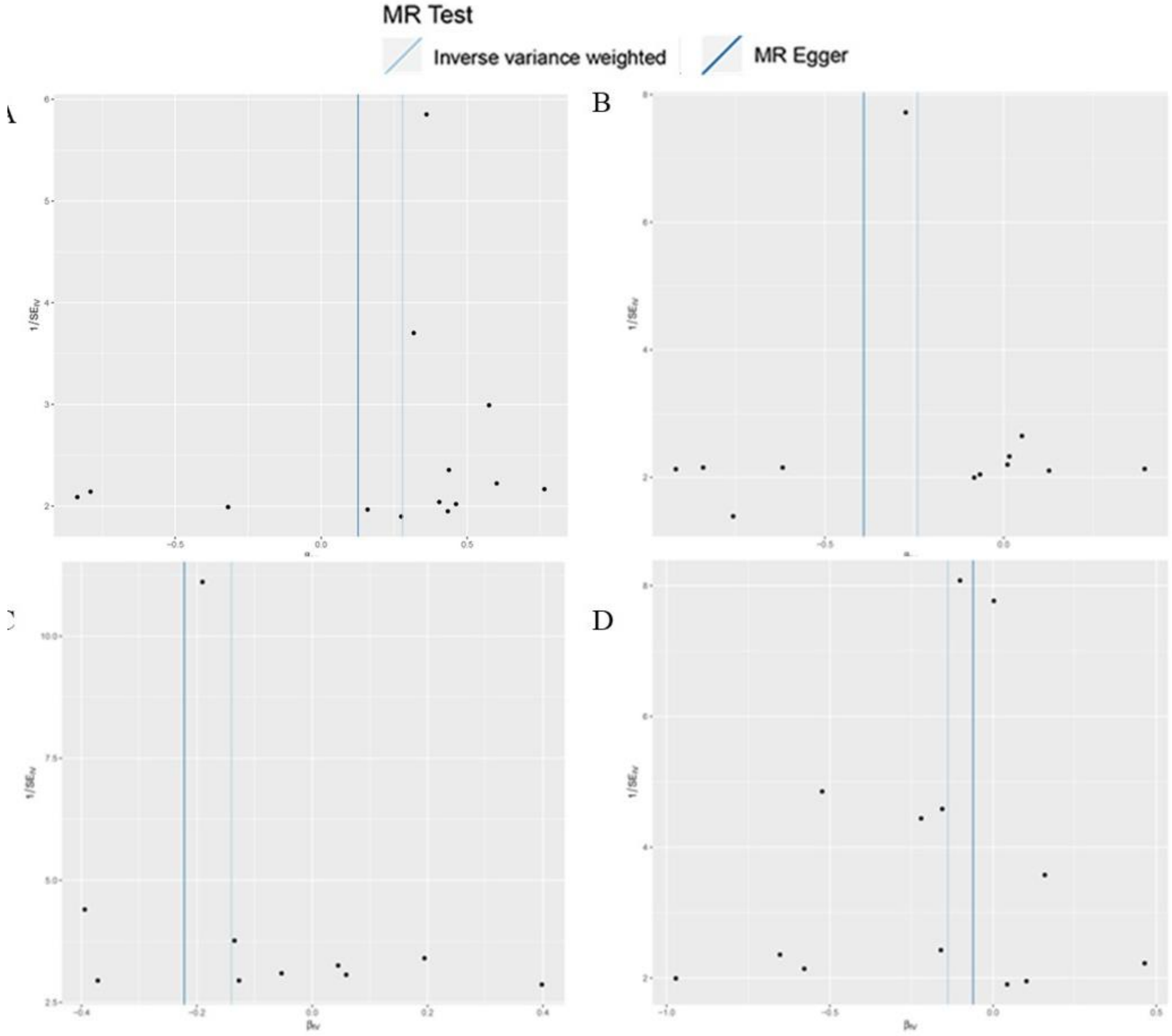

Figure S2

Funnel plots of Forward Mendelian randomization (MR) analyses for MCP-1 IL-10 IL-13 and TRAIL in squamous cell lung carcinoma which was applied to detect whether the observed association was along with obvious heterogeneity: (A) A funnel plot of the association between MCP-land squamous cell lung carcinoma. (B) A funnel plot of the association between IL-10 and squamous cell lung carcinoma. (C) A funnel plot of the association between IL-13 and squamous cell lung carcinoma.: (D) A funnel plot of the association between TRAIL and squamous cell lung carcinoma.

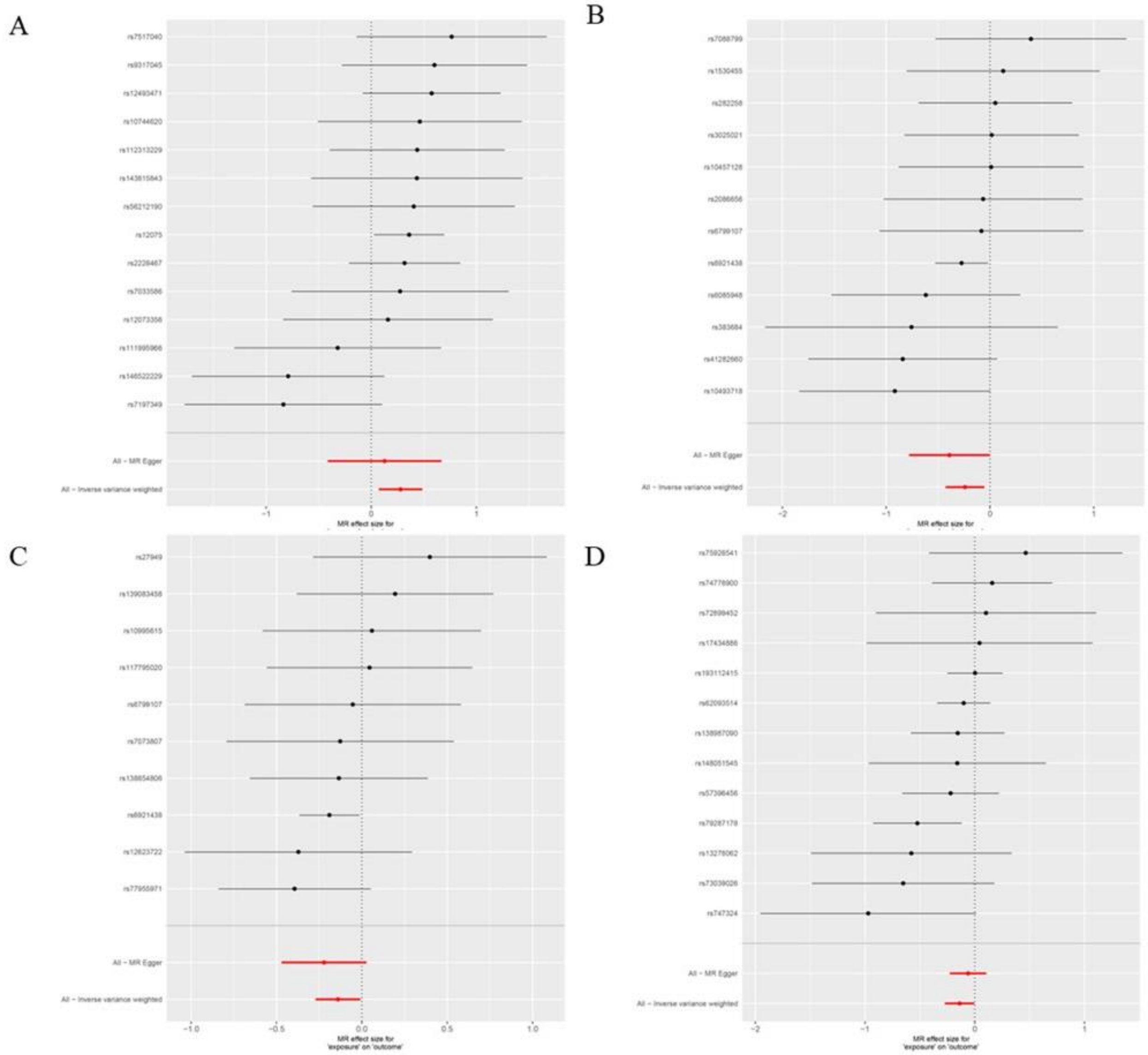

Figure S3

Forest plots of Forward Mendelian randomization (MR)analyses for MCP-1 IL-10 IL-13 and TRAIL in squamous cell lung carcinoma which was used to show the MR estimate and 95%CI values (gray line segment for each SNP which also shows the IVW and MR-Egger results at the bottom:(A) A forest plot of the association between MCP-land squamous cell lung carcinoma. (B) A forest plot of the association between IL-10 and squamous cell lung carcinoma. (C) A forest plot of the association between IL-13 and squamous cell lung carcinoma. (D) A forest plot of the association between TRAIL and squamous cell lung carcinoma.

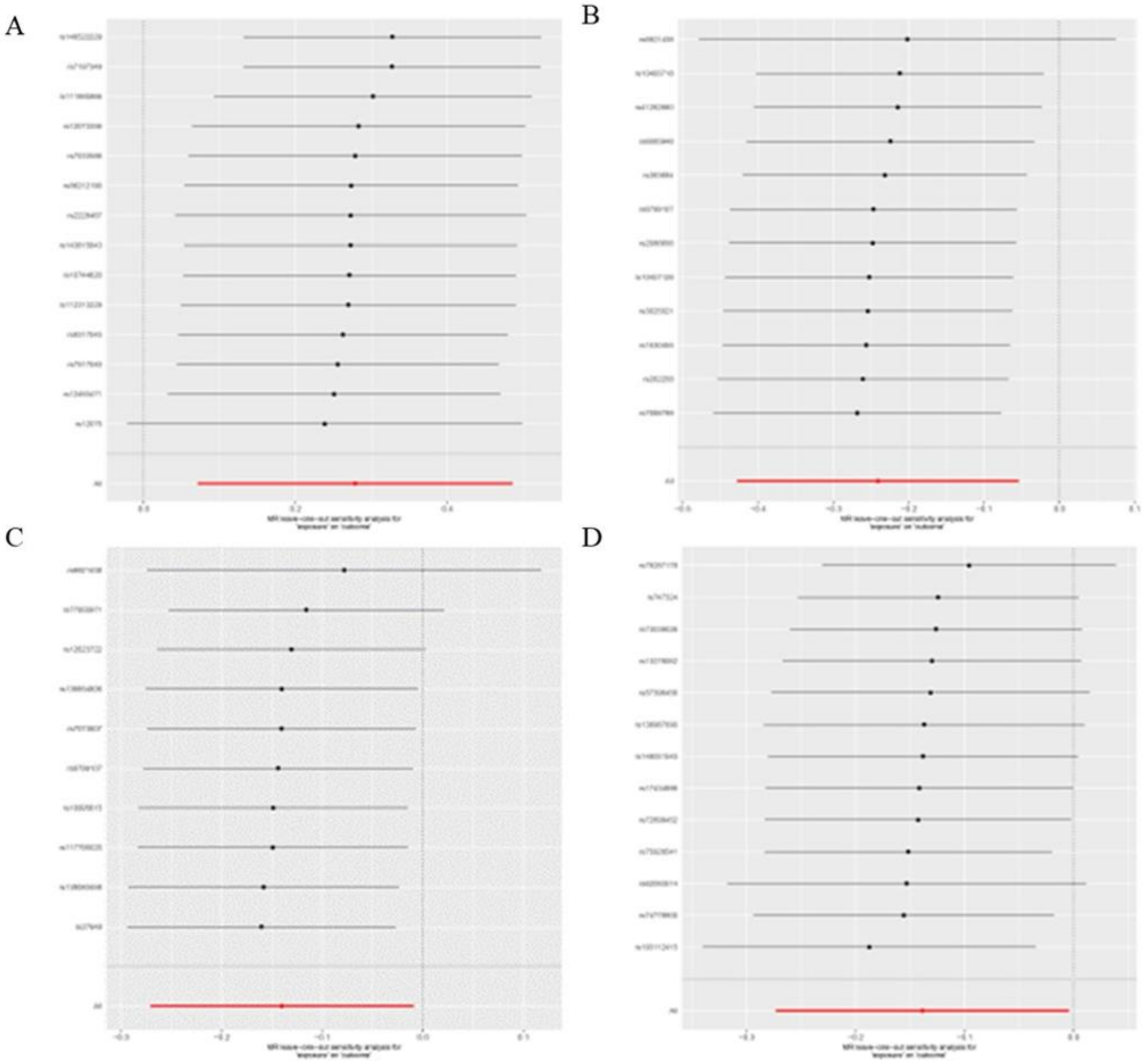

Figure S4

Leave-one-out analyses of Forward Mendelian randomization (MR) analyses for MCP-1, IL-10, IL-13 and TRAIL in squamous cell lung carcinoma to evaluate whether any single instrumental variable was driving the causal effect: (A) Leave-one-out analyses between MCP-1 and squamous cell lung carcinoma; (B) Leave-one-out analyses between IL-10 and squamous cell lung carcinoma; (C) Leave-one-out analyses between IL-13 and squamous cell lung carcinoma; (D)Leave-one-out analyses between TRAIL and squamous cell lung carcinoma.

Category	Exprosures	nSNPs	IVW.P	OR(95%CI)	Het.P	MR.PRESSO.P
Growth factors	B_NGF	5	0.4290	0.83 (0.52−1.32)	0.21	0.47
Chemokines	CTACK	14	0.0724	0.83 (0.67−1.02)	0.49	0.09
	EOTAXIN	14	0.9917	1.00 (0.77−1.30)	0.46	0.99
	FGF_BASIC	6	0.4013	1.42 (0.62−3.24)	<0.05	0.44
	G_CSF	8	0.5788	1.16 (0.68−1.97)	0.14	0.60
	GROA	8	0.5779	0.93 (0.73−1.20)	0.05	0.60
	HGF	6	0.3752	0.74 (0.37−1.45)	0.08	0.42
Other	IFN_G	9	0.7464	1.10 (0.61−1.98)	0.06	0.75
Interleukins	IL_10	12	0.9142	0.98 (0.72−1.34)	0.21	0.92
	IL_12_P70	14	0.9586	1.01 (0.80−1.27)	0.30	0.96
	IL_13	10	0.8315	1.02 (0.83−1.26)	0.30	0.84
	IL_16	9	0.8644	1.02 (0.82−1.27)	0.44	0.87
	IL_17	7	0.6478	1.16 (0.61−2.24)	<0.05	0.66
	IL_18	11	0.7480	0.96 (0.77−1.20)	0.87	0.67
	IL_1B	4	0.3211	1.26 (0.80−1.99)	0.82	0.17
	IL_1RA	7	0.7262	1.06 (0.75−1.51)	0.96	0.51
	IL_2	7	0.5150	1.10 (0.82−1.48)	0.93	0.29
	IL_2RA	6	0.2233	1.25 (0.87−1.78)	0.08	0.28
	IL_4	13	0.2128	0.79 (0.54−1.14)	0.83	0.14
	IL_5	8	0.4585	1.18 (0.77−1.80)	<0.05	0.48
	IL_6	9	0.9025	0.97 (0.55−1.69)	0.09	0.91
	IL_7	10	0.9002	0.99 (0.79−1.24)	0.35	0.90
	IL_8	7	0.7567	0.93 (0.60−1.45)	0.12	0.77
	IL_9	4	0.6014	1.14 (0.70−1.87)	0.56	0.57
	IP_10	9	0.7757	1.04 (0.78−1.39)	0.88	0.69
	M_CSF	9	0.8924	0.98 (0.71−1.35)	0.06	0.90
	MCP_1_MCAF	14	0.6601	0.94 (0.71−1.24)	0.47	0.66
	MCP_3	4	0.1623	0.83 (0.64−1.08)	0.88	0.06
	MIF	6	0.7717	0.95 (0.66−1.35)	0.81	0.68
	MIG	11	0.6644	1.06 (0.82−1.37)	0.88	0.56
	MIP_1A	4	0.5870	0.88 (0.56−1.38)	0.61	0.54
	MIP_1B	17	0.9293	0.99 (0.84−1.18)	0.16	0.93
	PDGF_BB	12	0.6526	0.94 (0.73−1.22)	0.79	0.59
	RANTES	8	0.3486	0.85 (0.60−1.20)	0.27	0.38
	SCF	9	0.2734	0.80 (0.53−1.20)	0.61	0.25
	SCGF_B	18	0.7007	0.96 (0.80−1.16)	0.53	0.70
	SDF_1A	7	0.7534	0.89 (0.43−1.84)	0.06	0.76
	TNF_A	5	0.6962	0.93 (0.63−1.36)	0.48	0.70
	TNF_B	4	0.2469	0.78 (0.51−1.19)	0.09	0.33
	TRAIL	13	0.8950	1.01 (0.84−1.22)	0.47	0.90
	VEGF	11	0.2972	1.10 (0.92−1.32)	0.51	0.30

Figure S5. Results of Forward Mendelian randomization of inflammatory cytokines and small cell lung cancer

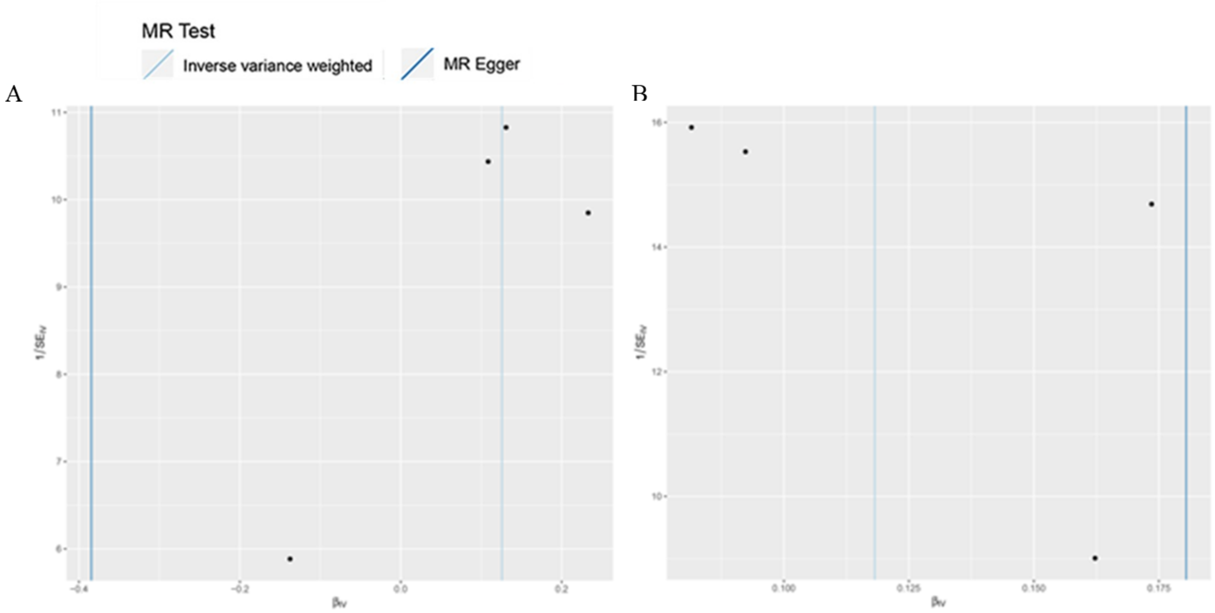

Figure S6

Funnel plots of Reverse Mendelian randomization (MR) analyses for B-NGF and SDF-1A in squamous cell lung carcinoma which was applied to detect whether the observed association was along with obvious heterogeneity: (A) A funnel plot of the association between B-NGF and squamous cell lung carcinoma; (B) A funnel plot of the association between SDF-1A and squamous cell lung carcinoma.

A B

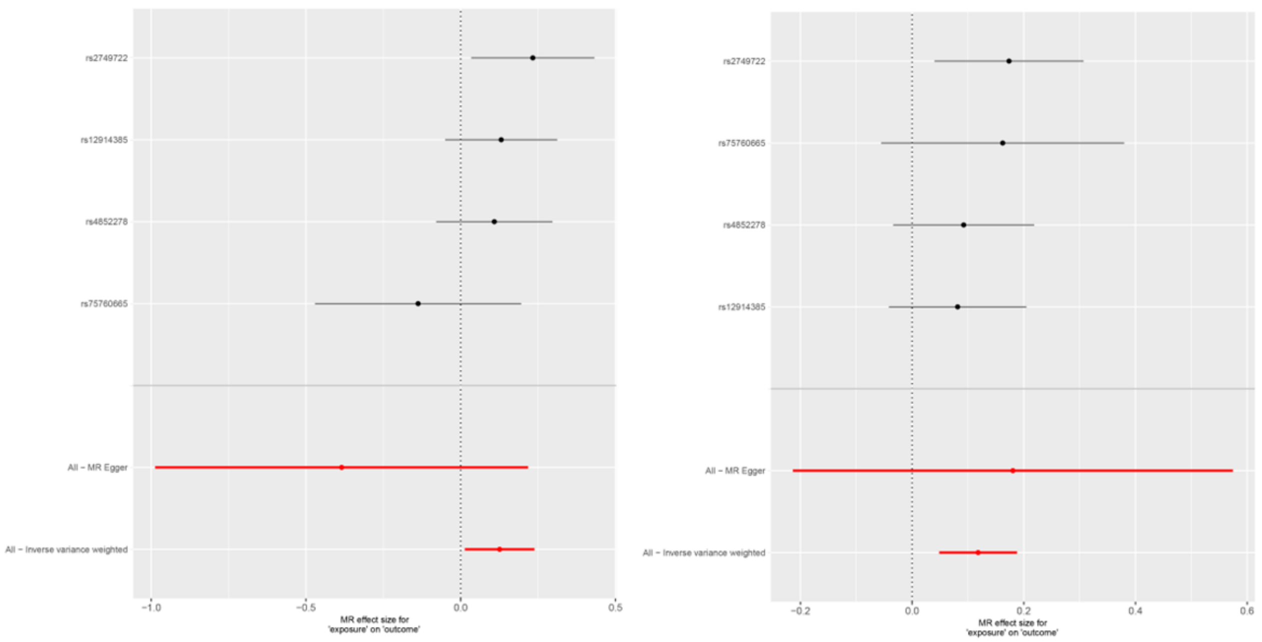

Figure S7

Forest plots of Reverse Mendelian randomization (MR) analyses for B-NGF and SDF-1A in squamous cell lung carcinoma used to show the MR estimate and 95%CI values (gray line segment) for each SNP which also shows the IVW and MR-Egger results at the bottom: (A) A Forest plot of the association between B-NGF and squamous cell lung carcinoma. (B) A Forest plot of the association between SDF-1A and squamous cell lung carcinoma.

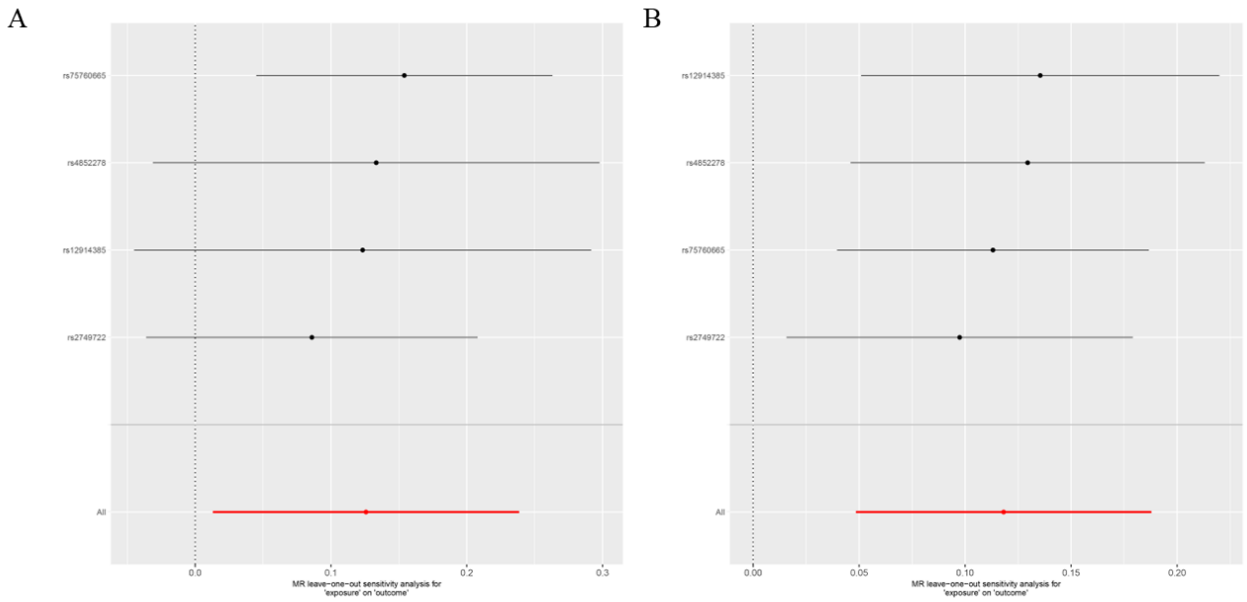

Figure S8

Leave-one-out analyses of Reverse Mendelian randomization (MR) analyses for B-NGF and SDF-A in squamous cell lung carcinoma to evaluate whether any single instrumental variable was driving the causal effect: (A) Leave-one-out analyses of the association between B-NGF and squamous cell lung carcinoma; (B) Leave-one-out analyses of the association between SDF-1A and squamous cell lung carcinoma.

Category	Outcomes	nSNPs	IVW.P	OR(95%CI)	Het.P	MR.PRESSO.P
Growth factors	B_NGF	6	0.8091	0.98 (0.84−1.14)	0.09	0.82
Chemokines	CTACK	6	0.2185	1.07 (0.96−1.19)	0.78	0.14
	EOTAXIN	6	0.3664	0.97 (0.90−1.04)	0.81	0.24
	FGF_BASIC	6	0.5155	1.03 (0.95−1.12)	0.31	0.54
	GROA	6	0.5139	0.96 (0.86−1.08)	0.80	0.38
	G_CSF	6	0.6867	0.98 (0.90−1.07)	0.20	0.70
	HGF	6	0.6485	0.97 (0.87−1.09)	<0.05	0.67
Other	IFN_G	6	0.6565	0.98 (0.90−1.07)	0.21	0.68
Interleukins	IL_10	6	0.8523	0.99 (0.92−1.07)	0.98	0.63
	IL_12_P70	6	0.2090	0.95 (0.89−1.03)	0.94	0.05
	IL_13	6	0.9234	1.01 (0.90−1.12)	0.96	0.84
	IL_16	6	0.2988	1.06 (0.95−1.18)	0.53	0.31
	IL_18	6	0.6353	0.97 (0.87−1.09)	0.37	0.66
	IL_1B	6	0.4210	0.95 (0.85−1.07)	0.38	0.46
	IL_1RA	6	0.1874	0.92 (0.82−1.04)	0.29	0.24
	IL_2RA	6	0.1226	1.11 (0.97−1.27)	0.16	0.18
	IL_2	6	0.5327	0.96 (0.84−1.09)	0.24	0.56
	IL_4	6	0.6452	0.98 (0.91−1.06)	0.48	0.65
	IL_5	6	0.8981	0.99 (0.89−1.11)	0.88	0.84
	IL_6	6	0.6286	0.98 (0.91−1.06)	0.93	0.39
	IL_7	6	0.4640	0.96 (0.85−1.08)	0.38	0.50
	IL_8	6	0.8720	0.99 (0.87−1.12)	0.28	0.88
	IL_9	6	0.6722	1.02 (0.92−1.14)	0.72	0.60
	IP_10	6	0.5723	0.96 (0.85−1.09)	0.25	0.60
	MCP_1_MCAF	6	0.4009	1.03 (0.96−1.11)	0.79	0.28
	MCP_3	6	0.5323	0.94 (0.77−1.14)	0.63	0.49
	MIF	6	0.5492	0.97 (0.87−1.08)	0.86	0.38
	MIG	6	0.5881	0.96 (0.84−1.11)	0.13	0.61
	MIP_1A	6	0.3495	1.05 (0.94−1.18)	0.95	0.11
	M_CSF	6	0.6738	1.03 (0.90−1.17)	0.74	0.60
	PDGF_BB	6	0.2821	0.96 (0.89−1.03)	0.91	0.11
	RANTES	6	0.4468	0.96 (0.86−1.07)	0.61	0.41
	SCF	6	0.9859	1.00 (0.93−1.07)	0.80	0.98
	SCGF_B	6	0.7913	1.03 (0.83−1.27)	<0.05	0.80
	SDF_1A	6	0.0595	0.93 (0.86−1.00)	0.92	0.02
	TNF_A	6	0.8844	0.99 (0.89−1.11)	0.62	0.87
	TNF_B	4	0.8749	1.02 (0.79−1.31)	0.15	0.88
	TRAIL	6	0.8770	1.01 (0.94−1.08)	0.53	0.87
	VEGF	6	0.9556	1.00 (0.92−1.08)	0.61	0.95

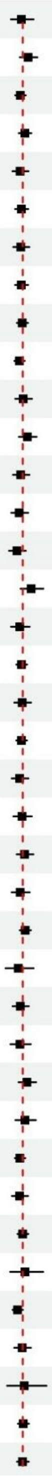

Figure S9. Results of Reverse Mendelian randomization of inflammatory cytokines and lung adenocarcinoma

Category	Outcomes	nSNPs	IVW.P	OR(95%CI)	Het.P	MR.PRESSO.P
Growth factors	B_NGF	8	0.8957	0.99 (0.90−1.09)	0.31	0.90
Chemokines	CTACK	8	0.7994	1.01 (0.92−1.11)	0.31	0.81
	EOTAXIN	8	0.8005	0.99 (0.94−1.05)	0.50	0.80
	FGF_BASIC	8	0.8787	1.00 (0.94−1.06)	0.85	0.83
	GROA	8	0.4095	0.94 (0.82−1.09)	<0.05	0.44
	G_CSF	8	0.5608	0.98 (0.93−1.04)	0.50	0.56
	HGF	8	0.2904	0.97 (0.92−1.03)	0.74	0.22
Other	IFN_G	8	0.5467	0.98 (0.93−1.04)	0.70	0.48
Interleukins	IL_10	8	0.5254	1.02 (0.96−1.08)	0.99	0.15
	IL_12_P70	8	0.4546	0.98 (0.92−1.04)	0.81	0.34
	IL_13	8	0.7220	0.98 (0.90−1.07)	0.78	0.65
	IL_16	8	0.7496	1.01 (0.93−1.11)	0.88	0.65
	IL_18	8	0.2046	1.06 (0.97−1.15)	0.53	0.22
	IL_1B	8	0.6858	1.02 (0.93−1.12)	0.35	0.70
	IL_1RA	8	0.5215	0.97 (0.89−1.06)	0.97	0.25
	IL_2RA	8	0.8751	0.99 (0.91−1.08)	0.55	0.87
	IL_2	8	0.4451	0.97 (0.89−1.05)	0.60	0.42
	IL_4	8	0.4583	0.98 (0.92−1.04)	0.76	0.37
	IL_5	8	0.6825	1.02 (0.93−1.11)	0.99	0.39
	IL_6	8	0.4866	0.98 (0.92−1.04)	0.89	0.32
	IL_7	8	0.4938	0.97 (0.89−1.06)	0.99	0.16
	IL_8	8	0.2929	0.95 (0.87−1.04)	0.87	0.16
	IL_9	8	0.2943	1.05 (0.96−1.14)	0.76	0.22
	IP_10	8	0.5042	0.97 (0.89−1.06)	0.57	0.48
	MCP_1_MCAF	8	0.9400	1.00 (0.94−1.06)	0.37	0.94
	MCP_3	7	0.7173	1.04 (0.86−1.25)	0.24	0.73
	MIF	8	0.2360	0.95 (0.87−1.04)	1.00	0.00
	MIG	8	0.2393	0.95 (0.87−1.03)	0.93	0.09
	MIP_1A	8	0.7061	1.02 (0.93−1.11)	0.65	0.67
	M_CSF	8	0.6811	0.97 (0.83−1.13)	<0.05	0.69
	PDGF_BB	8	0.2714	0.97 (0.91−1.03)	0.93	0.11
	RANTES	8	0.8670	0.99 (0.91−1.08)	0.95	0.77
	SCF	8	0.2093	0.96 (0.90−1.02)	0.33	0.25
	SCGF_B	8	0.2864	0.95 (0.88−1.04)	0.49	0.30
	SDF_1A	8	0.1618	0.96 (0.90−1.02)	0.90	0.06
	TNF_A	8	0.9326	1.00 (0.91−1.09)	0.68	0.92
	TNF_B	5	0.6205	0.96 (0.83−1.11)	0.48	0.62
	TRAIL	8	0.6472	1.02 (0.95−1.09)	0.17	0.66
	VEGF	8	0.8477	1.01 (0.95−1.07)	0.97	0.71

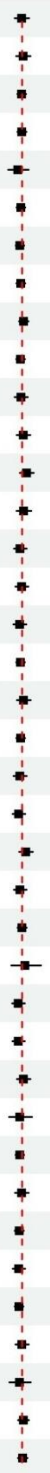

Figure S10. Results of Reverse Mendelian randomization of inflammatory cytokines and squamous cell lung carcinoma
